# Prevalence and genotypic–phenotypic association of highly resistant *Klebsiella pneumoniae* in community-acquired urinary tract infections

**DOI:** 10.1186/s12879-025-12071-2

**Published:** 2025-11-22

**Authors:** Nehal A. Dokmak, Salah Abdalla, Amr Tarek, Nesreen A. Safwat

**Affiliations:** 1 Department of Microbiology and Immunology, Faculty of Pharmacy, Modern University for Technology and Information, Cairo, Egypt; 2 Department of Microbiology and Immunology, Faculty of Pharmacy, University of Suez-Canal, Ismailia, Egypt

**Keywords:** Antimicrobial resistance, Egypt, *Klebsiella pneumoniae*, Resistance genes, Urinary tract infection, Virulence genes

## Abstract

**Introduction:**

Urinary tract infection (UTI) is a common infectious disease.

**Aim:**

To collect data on community-acquired UTI in Egyptians at 27 different cities in Egypt, including Cairo, to investigate the association between the phenotypic and genotypic characteristics of *Klebsiella pneumoniae* (*K. Pneumoniae*) as a highly virulent UTI pathogen.

**Methods:**

Collection of urine samples from different genders and ages. Isolation of bacteria by culturing samples on blood, MacConkey, and nutrient agar plates. Identification of *Klebsiella* spp based on cultural characteristics, Gram staining, and the DL microbial ID/AST automated system. Antimicrobial minimum inhibitory concentration (MIC) values of 100 *K. pneumoniae* isolates were determined using the DL microbial ID/AST automated system. Detection of Extended-Spectrum Beta-Lactamases (ESBL) producing *K. pneumoniae* strains using double disc synergy testing. Extraction of DNA from 49 *K. pneumoniae* isolates by an automated Zybio EXM 3000 extractor system. Detection of resistance and virulence genes using Polymerase Chain Reaction (PCR). Statistical analysis using SPSS version 23.

**Results:**

For the first time, a high prevalence of Pan Drug-Resistant (PDR) isolates (32.6%), 57.1% as Carbapenem-Resistant *K. pneumoniae* (CRKP), and 81.6% as ESBL-producers. Significant positive relationship in PDR *K. pneumoniae* with *bla-*_*NDM*_, *bla-*_*OXA−48*_, and *bla-*_*KPC*_, as well as CRKP with *bla-*_*NDM*_ (89%), *bla*_*− OXA−48*_ (32%), and *bla-*_*TEM*_ (75%). *rmpA* exhibited a significant positive relationship with PDR and CRKP. A positive relationship was exhibited between *bla*_*− NDM*_ and *bla-*_*OXA−48*_, *mrkD* and *fim-H*, and *bla*_*− CTXM*_ and *bla*_*− TEM*_.

**Conclusion:**

The prevalence of PDR *K. pneumoniae* was higher than previously reported. It is possible that some highly resistant *K. pneumoniae* strains also have increased pathogenic potential due to the observed positive associations between resistance genes and virulence genes. This relationship should be considered in developing treatment plans to ensure effectiveness against resistance and sufficient to neutralize virulence with minimal complications, using aggressive or combination therapy in the early stages.

**Clinical trial number:**

Not applicable.

**Supplementary Information:**

The online version contains supplementary material available at 10.1186/s12879-025-12071-2.

## Key summary points

UTIs are common and increasingly complicated by antibiotic-resistant pathogens.

There is limited data on community-acquired *K. pneumoniae* UTIs in Egypt, particularly regarding resistance and virulence profiles.

The study aimed to investigate the phenotypic and genotypic characteristics of *K. pneumoniae* from non-hospitalized Egyptian UTI patients and examine the association between resistance and virulence factors.

The significant relationship among resistance and virulence genes in *K. pneumoniae* underlines the critical need for genetic and antimicrobial resistance surveillance and targeted treatment options for community-acquired UTIs in Egypt.

**Supplementary Information:**

The online version contains supplementary material available at 10.1186/s12879-025-12071-2.

## Introduction

Urinary tract infections (UTIs) are prevalent, and bacteria-caused infections are present in healthcare and the community. Bacteria colonizing the urethra or periurethral area migrate into the bladder, causing an inflammatory reaction, which is the most common mode of infection. Although less frequently, other microorganisms, including fungi and certain viruses, have been reported to be responsible for UTIs [1, 2]. The most frequent UTI symptoms include urgency, frequency, and dysuria [3]. UTI is classified as an uncomplicated lower UTI, and it is one of the most treated infections in primary care. A complicated UTI is associated with a condition, such as a structural or functional abnormality of the genitourinary tract or the presence of an underlying disease, which increases the risk of a more serious outcome compared to an uncomplicated UTI [4]. UTIs are more common in women than men, with 50%-60% of women experiencing at least one over their lifetime [1, 5]. Risk factors for UTIs can be behavioral, anatomical, or genetic, varying according to the population and type of UTI [6]. In UTI infections, antibiotic treatment is frequently initiated empirically before the results of urine culture and susceptibility testing are known. Proper antibiotic treatment in UTI patients appears to shorten hospital stays, which benefits patient outcomes and reduces healthcare costs [7]. In 2019, there were 405 million cases of UTI worldwide, with 236,790 associated deaths, representing a 60% increase in cases and a 140% increase in fatalities over 1990 [8]. *K. Pneumoniae* is the second most common etiological agent in community-acquired UTIs [9]. According to the World Health Organization (WHO), 2024, *K. Pneumoniae* carbapenem-resistant was identified as the top pathogen to guide studies, research, and the production of new antibiotics [10]. Three major forms of carbapenemases came into existence, including class A or serine protease, which was primarily found in *K. pneumoniae*. *bla-*_*KPC*_ can break down penicillins, carbapenems, cephalosporins, and aztreonam [11]. The New Delhi metallo-β-lactamase (NDM) variant was discovered in 2009 in a *K. pneumoniae* strain from a Swedish visitor admitted to Delhi hospitals [12]. NDM is an example of Metallo-β-lactamase (MBL) that can break down most β-lactams (including carbapenems) but not monobactams [13], and oxacillinase (OXA-48-like) types [14]. *K. pneumoniae*, which produces Extended-Spectrum Beta-Lactamases (ESBL), is a multidrug-resistant organism that poses the greatest risk to human health [15]. The increased usage of antibiotics, particularly third-generation cephalosporins, has resulted in the spread of ESBL-producing bacteria. The TEM enzyme is a key mechanism for Gram-negative bacteria to withstand beta-lactam drugs. Cefotaximases (CTX-M-ases) are a new class of broad-spectrum plasmid beta-lactamases produced by many genera of the Enterobacteriaceae family, including *K. pneumoniae* [16]. The pathogenicity of *K. pneumoniae* bacteria is linked to various virulence factors that enable it to evade the host’s innate defense responses. *K. pneumoniae* pathogenicity factors include capsules, exopolysaccharides linked with mucoviscosity, lipopolysaccharides (LPSs), adhesins, and iron absorption mechanisms [17]. *K. pneumoniae* isolates have been reported to attach more strongly to human epithelial cells, most likely owing to fimbrial or non-fimbrial adhesins. Most clinical isolates of *K. pneumoniae* express type 1 (mannose-sensitive) and type 3 (mannose-resistant) fimbrial adhesins. Type 3 fimbrial adhesins can mediate *K.pneumoniae* attachment to various human cells, including endothelial cells and epithelial cells of the respiratory and urinary tracts. The *mrkD* protein plays a vital role in the *K. pneumoniae* binding to collagen molecules [18]. The *rmpA* gene in *K. pneumoniae* is linked to hypermucoviscosity [19]. The *uge* gene encoding uridine diphosphate galacturonate 4-epimerase is required for capsule and smooth lipopolysaccharide production [20]. The *gyrB* gene is a single-copy gene present in all bacteria that express the ATPase domain of DNA gyrase, an enzyme required for DNA replication [21].

## Materials and methods

### Sample collection

Urine samples were obtained in collaboration with the Royal laboratory (Cairo, Egypt), a mega medical laboratory that received urine specimens from various laboratories across Egypt. The collection took place over three months (March to May 2022) and included samples from patients with clinically identified urinary tract infection symptoms. The study was designed to represent both genders of seven age groups (< 1 year, 1–17 years, 18–30, 31–40, 41–50, 51–64 years, and ≥ 65 years old). For adults and toilet-trained children, the midstream portion of the urine stream was collected in a sterile container, and the remaining urine was disposed of. For infants younger than 1 year a professional nurse applied a sterile pediatric urine collection bag to the precleaned perineum and left it there for up to an hour, or until urination occurred. The samples collected were promptly refrigerated at a temperature of 4 °C and analyzed within 24 h of collection [22, 23].

### Inclusion criteria

The study comprised Egyptian non-hospitalized patients with UTI symptoms, including dysuria, frequent urination, and cloudy or red urine with a strong odor. A positive culture (colony count > or equal 10^5^ CFU/ml) confirmed a urinary tract infection (UTI). The study included only urine samples collected following the Centers for Disease Control and Prevention (CDC) ‘s collection criteria [24].

### Exclusion criteria

The study excluded non-Egyptian patients. This study excluded individuals who had previously participated, were taking antibiotics at the time of specimen collection, or were catheterized. To ensure accurate classification of community-acquired urinary tract infections, urine samples from patients with any hospital admission within the previous 14 days were excluded.

### Isolation of bacterial strains

As part of the routine diagnostic workflow at the Royal Laboratory (Cairo, Egypt), urine specimens were processed using standard microbiological techniques as recommended by the Clinical and Laboratory Standards Institute (CLSI) [25]. Freshly prepared blood agar, MacConkey agar, and nutrient agar plates (CM0007, Oxoid Ltd, Basingstoke, Hants, England) were used for bacterial isolation. Upon receipt, urine samples were gently mixed to ensure homogeneity. A plastic calibrated inoculating loop (Chongqing New World Trading, Chongqing, China) was used to transfer 1 µL of the urine specimen onto the agar surface, following the semi-quantitative streak plate method. The inoculum was evenly distributed over the plate surface using a sterile loop to facilitate discrete colony formation. Inoculated plates were incubated aerobically at 37 °C for 24 h. Colony-forming units (CFU) were enumerated, and the bacterial concentration was expressed as CFU/mL. A growth density of **≥** 1 × 10⁵ CFU/mL was considered indicative of significant bacteriuria consistent with urinary tract infection [26, 27].

### Identification of *Klebsiella* spp using phenotypic characterization

All positive cultures were tested to select *Klebsiella* spp based on their cellular morphology, cultural characteristics, Gram staining type, motility, as well as biochemical reactions as citrate, triple sugar iron, urease, indole, bile esculin, oxidase, and catalase tests [27].

### Confirmation of *Klebsiella pneumoniae* using the DL microbial ID/AST automated system

Confirmation of *K. pneumoniae* identification and antimicrobial minimum inhibitory concentration (MIC) determination as well as biochemical tests, were carried out using the DL-120E card as this card contain wells some of them specific for MIC determination and the other for biochemical tests in DL microbial ID/AST automated system as per the manufacturer’s instructions of DL microbial ID/AST automated system (Zhuhai DL Biotech Co., Ltd, China) as follows:

### a. Biochemical test in the DL microbial ID/AST automated system

A volume of 100 µl freshly prepared bacterial suspension equivalent to 0.5 McFarland standard was used to be injected into biochemical test wells of the DL-120E card (Zhuhai DL Biotech Co., Ltd, China). Two drops of sterile paraffin oil were added to wells specifically for anaerobic reactions. One drop of Voges Proskauer reagent A and one drop of Voges Proskauer reagent B were added to the Voges Proskauer test well. After 20 min, a drop of indole reagent was introduced into the indole well, followed by the addition of a drop of phenylalanine deaminase into its biochemical well. A few minutes later, the results of biochemical reactions are directly detected by producing a color change or by adding a chromogenic agent using the DL microbial ID/AST automated system (Zhuhai DL Biotech Co., Ltd, China).

### b. Antimicrobial MIC determination test in the DL microbial ID/AST automated system

A volume of 50 µl of bacterial suspension was added to Muller-Hinton broth (MH) and injected into the well specific for antimicrobial MIC determination test and growth control wells of the DL-120E card (Zhuhai DL Biotech Co., Ltd, China). The test card was covered with plastic film and incubated at 37 °C for 24 h. The MIC values were determined by measuring the produced turbidity. The absorbance values indicated positive or negative results for turbidity at different antibiotic concentrations. The MIC values were used to categorize the isolated *K. pneumoniae* into sensitive or resistant according to CLSI guidelines [28].

### Detection of ESBL-producing strains using the double disk synergy test (DDST)

ESBL production was detected using the DDST approach. To test selected 100 isolates of *K. pneumoniae*, a loop of the colony was chosen and emulsified in normal saline to create a suspension, adjusted to 0.5 McFarland turbidity standards, and swabbed aseptically on an MH agar plate. A disk containing amoxicillin-clavulanic acid (AMC) (20/10 µg) (Oxoid Ltd, Basingstoke, Hants, England) was placed in the center of the MH agar plate. Cefotaxime (30 µg) (Oxoid Ltd, Basingstoke, Hants, England) or ceftazidime (30 µg) (Oxoid Ltd, Basingstoke, Hants, England) was placed 30 mm apart on either side of the central disk. The plates were incubated for 24 h at 37 °C. Any increase or extension of the inhibition zone towards AMC (20/10 µg) (Oxoid Ltd, Basingstoke, Hants, England) is considered an ESBL producer [29].

### Classification of *Klebsiella pneumoniae* clinical isolates according to their resistance pattern

The collected isolates of *K. pneumoniae* were categorized into four groups according to their resistance profile as follows: Sensitive, which were sensitive to most antibiotics. Multi-Drug-Resistant (MDR): which was non-susceptibility to at least one agent in three or more antimicrobial categories. Extensively Drug-Resistant (XDR): defined as non-susceptibility to at least one agent in all but two or fewer antimicrobial categories (i.e., bacterial isolates remain susceptible to only one or two categories). Pan Drug-Resistant (PDR): defined as non-susceptibility to all agents in all antimicrobial categories [30].

### Extraction of DNA of *Klebsiella pneumoniae* isolates

Out of the previously mentioned 100 *K. pneumoniae* isolates, 49 were selected randomly, and total nucleic acid extraction for these 49 *K. pneumoniae* isolates was performed by an automated Zybio EXM 3000 extractor system using the bacterial nucleic acid extraction kit (BF-B-32) (Ozyme, France). All components in the kit were removed, and the 32-well plates were turned upside down to allow any liquid clinging to the aluminum film or well walls to collect at the bottom of the plates. The plates were left to stand for 3–5 min. Then, the aluminum film of the 32-well plates was carefully peeled back, and 15 µL of [Proteinase K] was added to the wells. A volume of 200 µL of pre-treated sample with pretreated solution was added to each well as required. Finally, the nucleic acid extraction process was initiated by turning on the extraction system.

### Detection of resistance and virulence genes of *Klebsiella pneumoniae* clinical isolates using polymerase chain reaction (PCR)

Selected virulence and resistance genes were detected. Briefly, a volume of 20 µL of PCR reaction mixture was prepared using 10 µL of cosmo PCR master mix (Willowfort, Birmingham, England), 0.5 µL of each forward and reverse primer (10 pmol), 1 µL of DNA template, and 8 µL of pyrogen-free water. The prepared mixture was used with the primers mentioned in (Table 1) for performing PCR cycling. The gene amplification started with an initiation temperature of 95.0˚C for 3 min, followed by 30 cycles at a denaturation temperature of 95.0˚C for 30 s. Annealing was performed at a temperature of 58˚C for 30 s for the detection of carbapenem resistance genes (*bla*_*− KPC*_, *bla-*_*OXA48*_, *bla-*_*NDM*_) and virulence genes (*rmpA*, *fimH*, *uge*, *gyr-B-2*, *mrkD*), while it was performed at 61˚C for 30 s for ESBL-associated genes (*bla-*_*CTXM*,_ *bla*_*− TEM*)_. The elongation step for all genes was performed at a temperature of 72.0˚C for 1 min and 30 s, with the final extension of DNA at 72.0˚C for 5 min [31].

The DNA fragment produced by PCR was loaded into the wells of the prepared 1.5% agarose (Simply Agarose, Simply Scientific Inc., Taiwan) gel containing ethidium bromide (Bio Basic Inc., Ontario, Canada) using a micropipette, 100 bp DNA ladder H3 RTU (Genedirex, United States) is a ladder with specific size markers that need to be added to one of the wells as a reference for downstream analysis. The gel electrophoresis apparatus (Cleaver) was used with an electric current at a voltage of 8 V/cm to run the gel through electrophoresis. Following electrophoresis, the separated DNA bands were visualized using a Gel Doc/UV transilluminator (Spectroline, Westbury, NY, USA) [32].

**Table 1:** Sequences of primers of resistance and virulence genes used for PCR reaction and their amplicon sizes

Gene	Primer Sequence (5’→3’)	Amplicon size	Annealing temperature	References
**Resistance Genes**				
*bla-*_*KPC*_	F: GTATCGCCGTCTAGTTCTGC	637	58˚C	[33]
	R: GGTCGTGTTTCCCTTTAGCC			
*bla-*_*CTXM*_	F: GACGATGTCACTGGCTGAGC	500	61˚C	[34]
	R: AGCCGCCGACGCTAATACA			
*bla-*_*TEM*_	F: ATCAGCAATAAACCAGC	516	61˚C	[31]
	R: CCCCGAAGAACGTTTTC			
*bla-*_*OXA−48*_	F: TTGGTGGCATCGATTATCGG	744	58˚C	[35]
	R: GAGCACTTCTTTTGTGATGGC			
*bla-*_*NDM*_	F: TGGCAGCACACTTCCTATC	488	58˚C	[35]
	R: AGATTGCCGAGCGACTTG			
**Virulence Genes**				
*rmpA*	F: ACTGGGCTACCTCTGCTTCA	535	58˚C	[36]
	R: CTTGCATGAGCCATCTTTCA			
*fimH*	F: GCCAACGTCTACGTTAACCTG	180	58˚C	[36]
	R: ATATTTCACGGTGCCTGAAAA			
*uge*	F: TCTTCACGCCTTCCTTCACT	535	58˚C	[37]
	R: GATCATCCGGTCTCCCTGTA			
*gyr-B-2*	F: TCCGGCGGTCTGCACGGCGT	1127	58˚C	[37]
	R: TTGTCCGGGTTGTACTCGTC			
*mrkD*	F: CCACCAACTATTCCCTCGAA	226	58˚C	[37]

### Statistical analysis studies

The chi-square test was utilized to examine the statistical association between resistance and virulence genes among various categories of *K. pneumoniae*. Fisher’s exact test was used for cell counts less than 5, and both tests were also used to evaluate the coexistence of each gene with other genes. The phi coefficient is used to assess the strength of the relationships, where a value of 0 indicates no association between the variables. In contrast, values closer to -1 or 1 indicate stronger negative or positive associations, respectively. If the *P*-value was less than or equal to 0.05, it was considered statistically significant. The data analysis was conducted using SPSS version 23 for Windows (IBM Corp., Armonk, NY).

### Ethical statement

This study was approved by the Institutional Review Board (IRB) of Suez Canal University (approval no. 202203m2). All procedures were conducted following the ethical standards of the institutional and/or national research committees and with the 1964 Helsinki declaration and its later amendments or comparable ethical standards. Informed consent was waived by the IRB due to the use of anonymized, routine clinical samples, following national regulations.

## Results

### Confirmation of *Klebsiella pneumoniae* using the DL microbial ID/AST automated system

Routine diagnostic work was performed at the mega medical laboratory (Royal laboratory, Cairo, Egypt) over three months from March to May 2022. 9,317 yielded positive bacterial cultures. *Klebsiella* spp accounted for 2,275 of these isolates. 100 isolates had been randomly selected and confirmed as *K. pneumoniae* using the DL microbial ID/AST automated system (Zhuhai DL Biotech Co., Ltd, China).

### Determination of minimum inhibitory concentration (MIC) of the tested antibiotics against *Klebsiella pneumoniae* isolates

Antimicrobial agents related to different classes were used for MIC determination and resistance pattern of the 100 clinical isolates of *K. pneumoniae* using the DL microbial ID/AST automated system (Zhuhai DL Biotech Co., Ltd, China).

The results showed that high resistance of *K. pneumoniae* isolates to ampicillin (92%), where the MIC ≥ 32 µg/ml. The resistance was decreased using combinations of ampicillin/sulbactam (69%) and tazobactam/piperacillin (57%). The tested isolates of *K. pneumoniae* showed a high resistance to cephalosporins (83%), where the MIC values ranged from 4 to ≥ 64 µg/ml. In addition, high resistance values were also determined against sulfamethoxazole/trimethoprim (76%), quinolones (54%), and Nitrofurantoin (52%). Moderate resistance of *K. pneumonia* isolates to both carbapenems and aminoglycosides was noticed, with percentages ranging from 33% to 43% (MIC values 2 µg/ml to ≥ 32 µg/ml and 8 µg/ml to ≥ 64 µg/ml, respectively) (Table S1).

### Classification of *Klebsiella pneumoniae* clinical isolates according to their resistance pattern

**Fig. 1:**
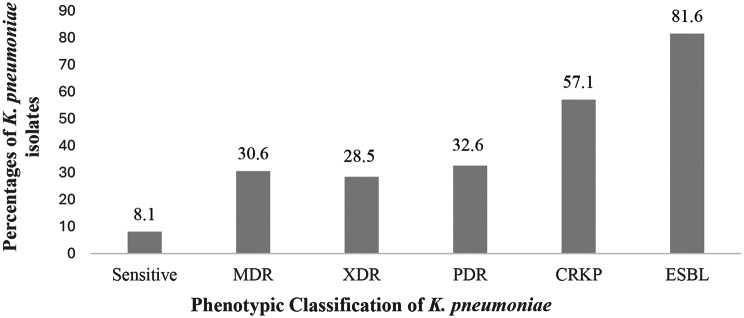
Distribution of Phenotypic Antimicrobial Resistance among *Klebsiella pneumoniae* Isolates. MDR: Multi-Drug-Resistant. XDR: Extensively Drug-Resistant. PDR: Pan Drug-Resistant. CRKP: Carbapenem-Resistant *Klebsiella pneumoniae*. ESBL: Extended-Spectrum Beta-Lactamases

The results showed that 8.1% (4/49) of *K pneumoniae* isolates were sensitive to most of the tested antibiotics, while 30.6% (15/49) were considered multidrug-resistant (MDR). A percentage of 28.5% (14/49) was considered (XDR). A relatively high percentage, 32.6% (16/49) of the tested isolates, were considered (PDR). In addition, A high percentage of 81.6% (40/49) of *K. pneumoniae* were considered ESBL-producers, and 57.1% (28/49) were (CRKP) (Fig. 1).

### Genotypic detection of resistance genes and virulence genes among *Klebsiella pneumoniae* isolates

The resistance genes of the selected 49 isolates of *K. pneumoniae*, including*bla*_*-CTXM*_, *bla*_*-TEM*_, *bla*_*-NDM*_, *bla*_*-OXA-48*_, and *bla*_*-KPC*,_ as well as virulent genes of *fim-H*, *mrkD*,* uge*, *gyr-B-2*, and *rmpA*, were investigated using PCR. The results of the prevalence of resistance & virulence genes (Tables 2 and 4).

The Amplification of *bla*_*-CTXM*_ (500 bp) and *bla*_*-TEM*_ (516 bp) was detected with a high percentage of 76% (37/49) and 63% (31/49), respectively. The results showed a high percentage of *bla*_*-NDM*_ of 51% (25/49) among the selected isolates, where its amplification was detected at an amplicon size of 488 bp. On the other hand, the lower predominance of genes *bla-*_*OXA-48*_ (744 bp) and *bla*_*-KPC*_ (637 bp) was demonstrated with low percentages of 18% (9/49) and 8% (4/49), respectively.

The most distributed virulence genes among *K. pneumoniae* were the *mrkD* and *fimH* genes, responsible for biofilm formation and adhesion, as both were detected in 98% (48/49) of the selected isolates. Additionally, the *uge* responsible for capsule synthesis and resistance to phagocytosis, as well as the g*yr-B-2* gene, important in DNA replication processes, were carried out by 37 out of 49(76%) of the tested isolates. Meanwhile, the *rmpA* gene was detected in only 10 (20%) of the tested isolates of *K. pneumoniae*.

Over half of the isolates carried ESBL genes *bla-*_*CTX−M*_ and *bla-*_*TEM*_. The *bla-*_*NDM*_ and *bla*_*− KPC*_ co-occurred with each ESBL gene at equal rates but in different isolates, whereas the virulence gene *rmpA* co-occurred with each ESBL gene in the same isolates.

All *bla*_*− OXA−48*_ positive isolates also carried *bla*_*− TEM*_ and *bla*_*− NDM*_. *fim-H* and *mrkD* were found in nearly all isolates and all carbapenem-resistant strains. The four isolates carrying *bla*_*− KPC*_ harbored *fim-H*, *uge*, *mrkD*, and *rmpA*, with *gyr-B-2* detected in one. All *rmpA* or *uge*-positive isolates also carried *fim-H* and *mrkD* (Table 2).

**Table 2:** Frequency of resistance & virulence genes and their coexistence patterns among the selected isolates of *Klebsiella pneumoniae*

Resistance Genes (*n* = 49)		Virulence Genes (*n* = 49)		
Gene	No (%)	Gene		No (%)
*bla-*_*CTXM*_	37(76%)	*fim-H*		48 (98%)
*bla-*_*TEM*_	31(63%)	*mrkD*		48 (98%)
*bla-*_*NDM*_	25(51%)	*uge*		37 (76%)
*bla-*_*KPC*_	4(8%)	*gyr-B-2*		37 (76%)
*bla-*_*OXA−48*_	9(18%)	*rmpA*		10 (20%)
**Co-existence of ESBL genes**				
**Gene**			**No (%)**	
*bla*_*− CTXM*_+ *bla*_*− TEM*_			27(55%)	
**Co-existence of ESBL genes + CR genes**				
**Gene**			**No (%)**	
*bla-*_*CTXM*_ *+bla-*_*NDM*_			21 (43%)	
*bla-*_*CTXM*_ *+bla-*_*KPC*_			1 (2%)	
*bla-*_*CTXM*_ *+bla-*_*OXA−48*_			7 (14%)	
*bla-*_*TEM*_ *+bla-*_*NDM*_			21 (43%)	
*bla-*_*TEM*_ *+bla-*_*KPC*_			1 (2%)	
*bla-*_*TEM*_ *+bla-*_*OXA−48*_			9 (18%)	
**Co-existence of CR encoding genes**				
**Gene**			**No (%)**	
*bla-*_*NDM*_ *+ bla-*_*KPC*_			3 (6%)	
*bla-*_*NDM*_ *+ bla-*_*OXA−48*_			9 (18%)	
*bla-*_*KPC*_ *+ bla-*_*OXA−48*_			1 (2%)	
**Co-existence of ESBL genes + Virulence genes**				
**Gene**			**No (%)**	
*bla-*_*CTXM*_ *+fim-H*			36 (73%)	
*bla-*_*CTXM*_ *+mrkD*			36 (73%)	
*bla-*_*CTXM*_ *+uge*			25(51%)	
*bla-*_*CTXM*_ *+gyr-B-2*			29 (59%)	
*bla-*_*CTXM*_ *+rmpA*			7 (14%)	
*bla-*_*TEM*_ *+fim-H*			30 (61%)	
*bla-*_*TEM*_ *+mrkD*			30 (61%)	
*bla-*_*TEM*_ *+uge*			20 (41%)	
*bla-*_*TEM*_ *+gyr-B-2*			22 (45%)	
*bla-*_*TEM*_ *+rmpA*			7 (14%)	
**Co-existence of CR genes + Virulence genes**				
**Gene**			**No (%)**	
*bla-*_*NDM*_ *+ fim-H*			25(51%)	
*bla-*_*NDM*_ *+ mrkD*			25(51%)	
*bla-*_*NDM*_ *+ uge*			16 (33%)	
*bla-*_*NDM*_ *+ gyr-B-2*			19 (39%)	
*bla-*_*NDM*_ *+rmpA*			9 (18%)	
*bla-*_*KPC*_ *+ fim-H*			4(8%)	
*bla-*_*KPC*_ *+ mrkD*			4(8%)	
*bla-*_*KPC*_ *+ uge*			4(8%)	
*bla-*_*KPC*_ *+ gyr-B-2*			1 (2%)	
*bla-*_*KPC*_ *+ rmpA*			4(8%)	
*bla-*_*OXA−48*_ *+fim-H*			9(18%)	
*bla-*_*OXA−48*_ *+mrkD*			9(18%)	
*bla-*_*OXA−48*_ *+uge*			6 (12%)	
*bla-*_*OXA−48*_ *+gyr-B-2*			8 (16%)	
*bla-*_*OXA−48*_ *+rmpA*			4(8%)	
**Co-existence of Virulence genes**				
**Gene**			**No (%)**	
*fim-H + mrkD*			48 (98%)	
*fim-H + uge*			37(76%)	
*fim-H + gyr-B-2*			36 (73%)	
*fim-H + rmpA*			10 (20%)	
*mrkD + uge*			37(76%)	
*mrkD + gyr-B-2*			36 (73%)	
*mrkD + rmpA*			10 (20%)	
*uge + gyr-B-2*			27 (55%)	
*uge + rmpA*			7 (14%)	
*gyr-B-2 + rmpA*			6 (12%)	

ESBL: Extended-Spectrum Beta-Lactamases. CR: Carbapenem-Resistant genes

### Association between phenotypic resistance, resistance, and virulence genes among the tested *Klebsiella pneumoniae* isolates

A high prevalence of *bla*_*− CTXM*_ was recorded in both MDR and XDR isolates of *K. pneumoniae* (80% and 86%, respectively). The prevalence of the *bla-*_*TEM*_ gene was positively associated with XDR isolates of *K. pneumoniae* (*P* value equal 0.039), while it was negatively associated with sensitive isolates (0%). Among PDR isolates of tested *K. pneumoniae*, a high prevalence of the *bla-*_*NDM*_ gene *(*88%) was recorded. *bla*-_*NDM*_, *bla*-_*OXA−48*,_ and *bla*-_*KPC*_ were significantly associated with phenotypic PDR isolates of *K. pneumoniae* (*P* value ≤ 0.05) (Table 3**).**

Significant positive association of *bla-*_*NDM*_, *bla-*_*OXA−48*_, and *bla*-_*TEM*_ genes within CRKP, with a high prevalence percentage of 89%, 32%, and 75%, respectively.

The results revealed that the *fim-H* and *mrkD* virulence genes were the most predominant in all resistant phenotypes of the tested *K. pneumoniae* strains (100% in MDR, PDR, and 93% in XDR). All tested virulence genes, except the *rmpA* gene, were detected with high prevalence (100%) in the sensitive isolates of *K. pneumoniae*. On the other hand, the *rmpA* displayed a significantly positive relationship with PDR as well as CRKP (*P* value ≤ 0.05). A negative relationship was reported between the *uge* gene and PDR phenotypic *K. pneumoniae* strains, as well as the *rmpA* gene and MDR isolates (*P* value ≤ 0.05). The *gyr-B-2* gene showed a high prevalence in all sensitive and resistant phenotypic *K. pneumoniae* strains, including CRKP (Table 3).

**Table 3:** Prevalence and association of resistance and virulence genes among different categories of *Klebsiella pneumoniae* isolates

Resistance Genes	The prevalent percentage of resistance genes in:								
	Sensitive	MDR		XDR		PDR		CRKP	
*bla-*_*CTXM*_	**0(0%)** ***P*** **= 0.002** ^b*^	12(80%)		12(86%)		13(81%)		23(82)	
*bla-*_*TEM*_	**0(0%)** ***P*** **= 0.014** ^b*^	7(47%)		**12(85%)** ***P*** **= 0.039** ^a*^		12(75%)		**21(75%)** ***P*** **= 0.049** ^a*^	
*bla-*_*CTXM*_ *+ bla-*_*TEM*_	**0(0%)** ***P =*** **0.035**^b*^	**5(33%)** ***P*** **= 0.042** ^b*^		10(71%)		12(75%)		**19(68%)** ***P*** **= 0.038**^a*^	
*bla-*_*NDM*_	**0(0%)** ***P*** **= 0.05** ^b*^	**0(0%)** ***P*** **= 0.000**^b*^		**11(79%)** ***P*** **= 0.015** ^a*^		**14(88%)** ***P*** **= 0.000** ^a*^		**25(89%)** ***P =*** **0.000**^a*^	
*bla-*_*KPC*_	0(0%)	0(0%)		0(0%)		**4(25%)** ***P*** **= 0.009** ^a*^		4(14%)	
*bla-*_*OXA 48*_	0(0%)	**0(0%)** ***P*** **= 0.042** ^b*^		3(21%)		**6(38%)** ***P*** **= 0.043** ^a*^		**9(32%)** ***P*** **= 0.006**^a*^	
**Virulence genes**	**The prevalent percentage of virulence genes in the** ***K. pneumoniae*** **categories**								
	**Sensitive**		**MDR**		**XDR**		**PDR**		**CRKP**
*fim-H*	4(100%)		15(100%)		13(93%)		16(100%)		28(100%)
*uge*	4(100%)		13(87%)		11(79%)		**9(56%)** ***P*** **= 0.04** ^b*^		19(68%)
*rmpA*	0(0%)		**0(0%)** ***P*** **= 0.021** ^b*^		3(21%)		**7(44%)** ***P*** **= 0.008** ^a*^		**10(36%)** ***P*** **= 0.003**^a*^
*mrkD*	4(100%)		15(100%)		13(93%)		16(100%)		28(100%)
*gyr-B-2*	4(100%)		11(73%)		10(71%)		12(75%)		21(75%)

Data from Statistical analysis is presented as *n* (%). The Chi-square test was performed to assess the association between resistance and virulent genes across *K. pneumoniae* categories, and the strength of these associations was measured using the Phi coefficient. Statistical significance was considered at *P* ≤ 0.05, and significant values are shown in bold. A positive relationship is denoted by (a*), while a negative relationship is indicated by (b*)

MDR: Multi-Drug-Resistant. XDR: Extensively Drug-Resistant. PDR: Pan Drug-Resistant. CRKP: Carbapenem-Resistant *Klebsiella pneumoniae*. ESBL: Extended-Spectrum Beta-Lactamases

### Prevalence of resistance genes among Extended-Spectrum Beta-Lactamases (ESBL) producing *Klebsiella pneumoniae* isolates

**Fig. 2:**
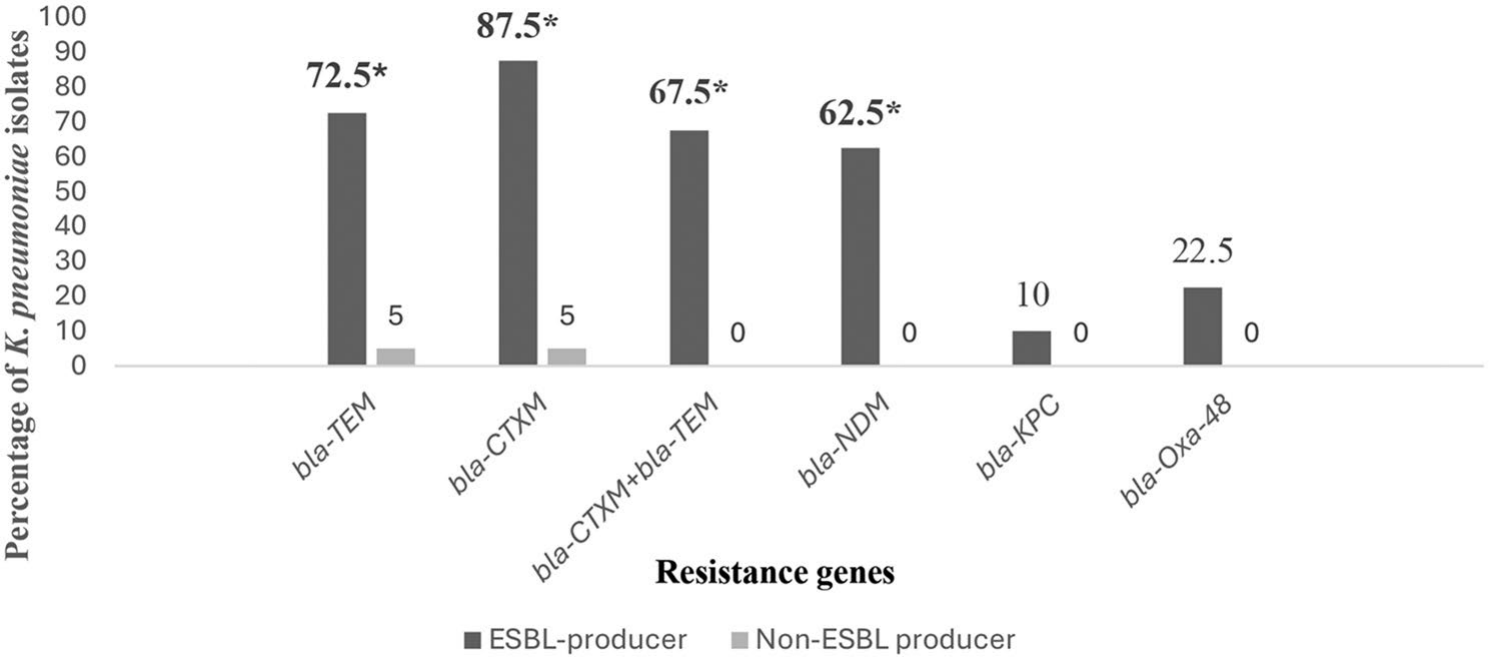
Distribution of resistance genes among ESBL-producing and non-ESBL-producing *Klebsiella pneumoniae* Isolates. Data are presented as **(%)**. Statistical significance was considered at *P* ≤ 0.05. Statistically significant and positive relationships are shown in **bold**. A positive relationship is indicated by (%)*. The Chi-square test was used to evaluate associations between resistance genes and the ESBL-producing phenotype of *K. pneumoniae*, and the phi coefficient was applied to determine the strength of these associations ESBL: Extended-Spectrum Beta-Lactamases. Non-ESBL: Non-Extended-Spectrum Beta-Lactamases

The results revealed that the most predominant resistant genes in ESBL-producer isolates of *K. pneumoniae* were *bla-*_*CTXM*_ (87.5%) and *bla-*_*TEM*_ (72.5%) (Fig. 2). In addition, the co-existence of both *bla-*_*CTXM*_ and *bla-*_*TEM*_ resistance genes was significantly positive among ESBL producer isolates of the tested *K. pneumoniae* (*P* value ≤ 0.05).

### Prevalence of virulence genes among ESBL and non-ESBL producers of *Klebsiella pneumoniae*

**Fig. 3:**
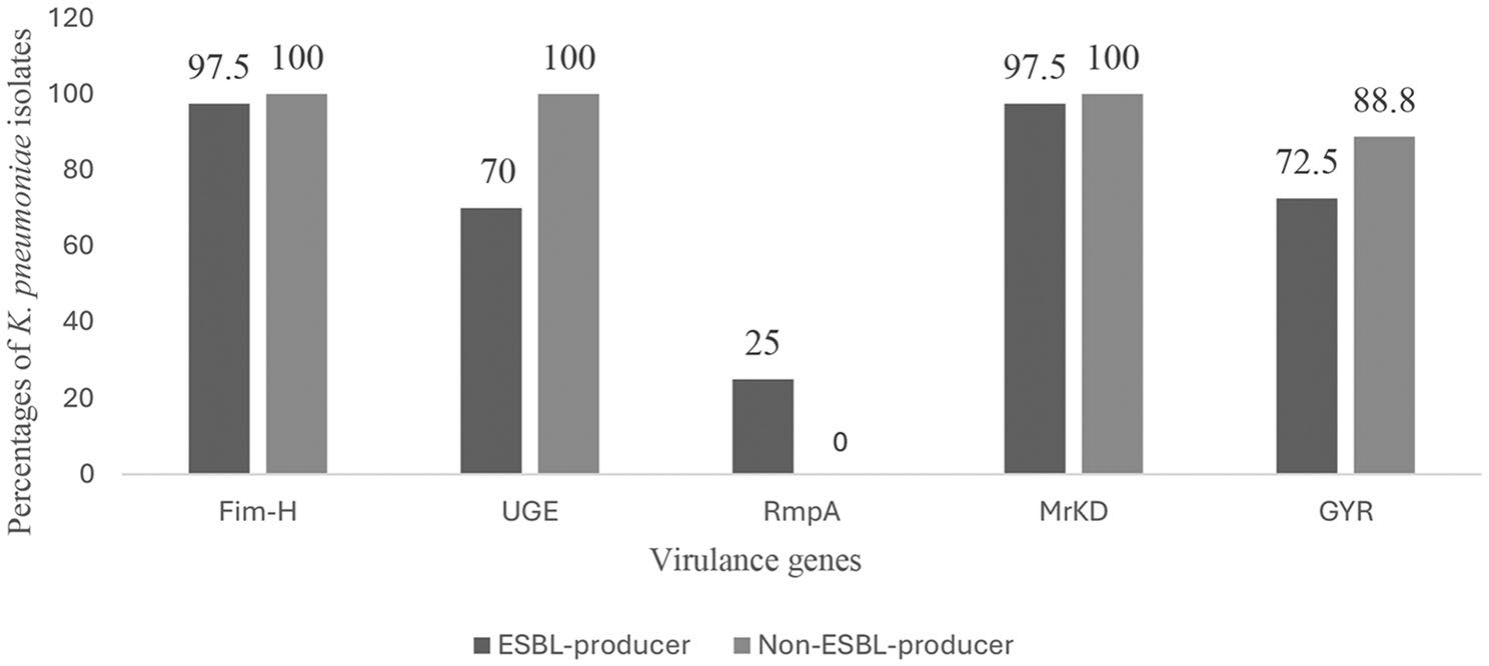
Distribution of virulence genes among ESBL-producing and non-ESBL-producing *Klebsiella pneumoniae* isolates. Data are presented as percentages (%). ESBL: Extended-Spectrum Beta-Lactamases. Non-ESBL: Non-Extended-Spectrum Beta-Lactamases

The results showed a high prevalence of the virulent genes among both ESBL and non-ESBL-producing *K. pneumoniae* isolates. The prevalence of the virulence genes among the ESBL-producing *K. pneumoniae* isolates is *fim-H* (97.5%) and *mrkD* (97.5%), followed by *gyr-B-2* (72.5%) and *uge* (70%). The *rmpA* gene was detected in a percentage of 25% in ESBL producers and was completely absent in non-ESBL producer isolates (Fig. 3).

### Resistance phenotypes, ESBL producers, resistance, and virulence genes among the tested genders and age groups

The findings revealed that the female gender had the highest prevalence of sensitive phenotypic (100%) and resistance phenotypic strains of MDR and PDR (73% and 56%, respectively). In contrast, the XDR *K. pneumoniae* strains were widely distributed among males (57%) more than among females (43%). The female gender had the highest prevalence of ESBL-associated resistance genes of *bla-*_*CTX−M*_ and *bla*_*− TEM*_, with a percentage of 54% and 58%, respectively, while males were highly affected by *K. pneumoniae* with resistance genes of *bla*_*− NDM*_ and *bla*-_*OXA−48*_ (56%). A high prevalence of *K. pneumoniae* with *bla-*_*KPC*_ gene resistance was observed among both genders, with the same percentage. The age group ranging from 18 to 30 had a high prevalence of phenotypic resistance strains (sensitive, XDR, and PDR), ESBL-producer strains, as well as all virulence genes, which were detected among the isolated *K. pneumoniae*, except for *rmpA.* All virulence genes were highly distributed among females, except for *rmpA*, which was substantially distributed in males, with a percentage of 60% (Table 4).

**Table 4:** Distribution of resistance phenotypes, ESBL production, resistance, and virulence genes among the tested genders and age groups

Variables		Prevalence of variables No. (%)									
		Gender		Age groups (Years)							
		Male	Female	< 1	1–17	18–30	31–40	41–50	51–64	> 64	
Phenotypic resistance	Sensitive	0 (0%)	4 (100%)	0(0%)	1 (25%)	2 (50%)	1 (25%)	0 (0%)	0 (0%)	0 (0%)	
	MDR	4 (27%)	11 (73%)	0 (0%)	0 (0%)	3(20%)	5(33%)	2 (13%)	2 (13%)	3 (20%)	
	XDR	8 (57%)	6 (43%)	3 (21%)	3 (21%)	3 (21%)	1 (8%)	0 (0%)	2 (14%)	2 (14%)	
	PDR	7 (44%)	9 (56%)	4 (25%)	2 (13%)	4 (25%)	0 (0%)	2 (13%)	1(6%)	3 (19%)	
ESBL producer		18 (45%)	22 (55%)	7 (18%)	5 (13%)	9(23%)	6 (18%)	3(8%)	3(8%)	7 (18%)	
CR and ESBL genes	*bla-*_*CTXM*_	17 (46%)	20 (54%)	6 (16%)	3 (8%)	10 (27%)	6(16%)	3(8%)	2(5%)	7 (19%)	
	*bla-*_*TEM*_	13 (42%)	18 (58%)	7 (23%)	4 (13%)	8 (26%)	3(10%)	2(6%)	2(6%)	5 (16%)	
	*bla-*_*NDM*_	14 (56%)	11 (44%)	6 (24%)	5 (20%)	7 (28%)	0 (0%)	1(4%)	3(12%)	3 (12%)	
	*bla-*_*KPC*_	2 (50%)	2 (50%)	0 (0%)	1 (25%)	0 (0%)	0 (0%)	1 (25%)	1 (25%)	1 (25%)	
	*bla-*_*OXA−48*_	5 (56%)	4 (44%)	3 (33%)	1 (11%)	3 (33%)	0 (0%)	1 (11%)	0 (0%)	1 (11%)	
Virulence genes	*fim-H*	19 (40%)	29 (60%)	7 (15%)	6 (13%)	12 (25%)	6 (13%)	4(8%)	5(10%)	8 (17%)	
	*uge*	14 (38%)	23 (62%)	3 (8%)	5 (14%)	9 (24%)	6(16%)	4(11%)	5 (14%)	5 (14%)	
	*rmpA*	6 (60%)	4 (40%)	3 (30%)	1 (10%)	2 (20%)	0 (0%)	1 (10%)	2 (20%)	1 (10%)	
	*mrkD*	19 (40%)	29 (60%)	7 (15%)	6 (13%)	12 (25%)	6 (13%)	4(8%)	5(10%)	8 (17%)	
	*gyr-B-2*	14 (38%)	23 (62%)	6 (16%)	4 (11%)	10 (27%)	6(16%)	3(8%)	4 (11%)	4 (11%**)**	

Data are presented as numbers (*n*) and percentages (%). Gender was categorized as male and female, while age groups were classified as < 1 year, 1–17 years, 18–30 years, 31–40 years, 41–50 years, 51–64 years, and > 64 years

MDR: Multi-Drug-Resistant. XDR: Extensively Drug-Resistant. PDR: Pan Drug-Resistant. CR: Carbapenem-Resistant. CRKP: Carbapenem-Resistant *Klebsiella pneumoniae*. ESBL: Extended-Spectrum Beta-Lactamase

### Association between resistance genes and virulence genes among the tested *Klebsiella pneumonia*e

A statistical association between resistance genes and virulence genes, as well as the association between both resistance and virulence genes among the tested isolates of *K. pneumoniae*, was performed. A substantial positive relationship between the ESBL-associated genes *bla-*_*CTXM*_ and *bla-*_*TEM*_ (*P* ≤ 0.05). *bla-*_*CTXM*,_ which has a substantial negative relationship with the carbapenemase gene *bla-*_*KPC*_ (*P* ≤ 0.05). The gene *bla-*_*TEM*_ has a significant positive relationship with the other carbapenemase genes *bla-*_*NDM*_ and *bla*_*− OXA−48*_. There was a significant relationship between the carbapenemase genes (*bla-*_*NDM*_ and *bla-*_*KPC*_) with the virulence gene *rmpA*, responsible for the regulator of mucoid phenotype A (*P* ≤ 0.05). The virulence genes *mrkD* and *fim-H* had a significant positive relationship with each other. On the other hand, a negative relationship of *bla-*_*CTXM*_ and *bla-*_*TEM*_ with the virulence gene *uge*, responsible for capsule synthesis and resistance to phagocytosis (*P* ≤ 0.05) (Table 5**)**.

**Table 5:** Coexistence of resistance and virulence genes and their association among the tested isolates of *Klebsiella pneumoniae*

Genes		Resistance genes					Virulence genes				
		*bla-*_*CTXM*_ *N* = 37	*bla-*_*TEM*_ *N* = 31	*bla-*_*OXA−48*_ *N* = 9	*bla-*_*NDM*_ *N* = 25	*bla-*_*KPC*_ *N* = 4	*rmpA* *N* = 10	*fim-H* *N* = 48	*uge* *N* = 37	*gyr-B-2* *N* = 37	*mrkD* *N* = 48
**Resistance genes**	*bla-*_*CTXM*_ *N* = 37	-	**27** ***P*** **= 0.019**^a*^	7	21	**1** ***P*** **= 0.041**^b*^	7	36	**25** ***P*** **= 0.024**^b*^	29	36
	*bla-*_*TEM*_ *N* = 31	-	-	**9** ***P*** **= 0.018**^a*^	**21** ***P*** **= 0.002**^a*^	1	7	30	**20** ***P*** **= 0.036**^b*^	22	30
	*bla-*_*OXA−48*_ *N* = 9	-	-	-	**9** ***P*** **= 0.002**^a*^	**1**	4	9	6	8	9
	*bla-*_*NDM*_ *N* = 25	-	-	-	-	3	**9** ***P*** **= 0.011**^a*^	25	16	19	25
	*bla-*_*KPC*_ *N* = 4	-	-	-	-	-	**4** ***P*** **= 0.001**^a*^	4	4	**1** ***P*** **= 0.041**^b*^	4
**Virulence genes**	*rmpA* *N* = 10	-	-	-	-	-	-	10	7	6	10
	*fim-H* *N* = 48	-	-	-	-	-	-	-	37	36	**48** ***P*** **= 0.02**^a*^
	*uge* *N* = 37	-	-	-	-	-	-	-	-	27	37
	*gyr-B-2* *N* = 37	-	-	-	-	-	-	-	-	-	36
	*mrkD* *N* = 48	-	-	-	-	-	-	-	-	-	-

The data represent the coexistence of resistance and virulence genes detected in *K. pneumoniae* isolates. Statistical significance was considered at *P* ≤ 0.05 and shown in bold. A positive relationship is indicated by (a*), while a negative relationship is indicated by (b*). Fisher’s exact test was applied for cell counts less than 5, and both the Chi-square and Fisher’s tests were used to assess the coexistence of each gene with others. The phi coefficient was used to measure the strength of these associations

## Discussion

Over the past decades, the widespread emergence of resistant pathogens, along with highly virulent strains, has triggered numerous public health crises. In the case of *K. pneumoniae*, it can cause a wide range of community-acquired urinary tract infections among both genders of different ages [36]. The rise of resistant uropathogens has decreased therapy choices for UTIs globally, potentially leading to treatment failures [38]. This study highlights the high prevalence of *K. pneumoniae* that are highly resistant to the most common antibiotics used in the Egyptian community in association with virulence genotypes.

Antimicrobial agents related to different classes were used for MIC determination and resistance pattern design of the 100 clinical isolates of *K. pneumoniae*. Interestingly, third-generation cephalosporins, which are often prescribed for empirical treatment of UTIs, exhibited a markedly higher proportion of resistance among *K. pneumoniae* isolates compared to nitrofurantoin, which agreed with a study conducted in Iran [39]. In contrast, nitrofurantoin, an older, low-cost, urinary tract infection-specific antimicrobial, retained substantially higher activity against the isolates. This finding underscores the potential clinical value of reconsidering nitrofurantoin as a first-line therapeutic option for uncomplicated UTIs, particularly in the context of rising resistance to broad-spectrum cephalosporins. Such an approach could improve treatment efficacy while reducing selective pressure on higher-tier antibiotics, aligning with antimicrobial stewardship principles.

Our results showed a notably high prevalence of PDR (32.6%), MDR (30.6%), and XDR (28.5%) *K. pneumoniae* was recorded, marking, to our knowledge, the first report of PDR dominance in Egypt. The higher incidence of MDR *K. pneumoniae* in our study matches many other national and global studies [40, 41]. This alarming sign could be attributed to a lack of newer antimicrobials, limited adherence to infection control protocols, and unnecessary use of antimicrobials.

As carbapenem-resistant and ESBL-producing Enterobacteriaceae are on the WHO priority pathogens list for research and development of new antibiotics [42]. We assessed their prevalence among the tested clinical isolates of *K. pneumoniae*.

The results revealed that carbapenem-resistant *K. pneumoniae* (CRKP), ESBL-producing isolates were highly prevalent (57.1% and 81.6% respectively), exceeding rates reported in earlier national studies, where a lower percentage of ESBL-producing *K. pneumoniae* incidence (34.4%) and CRKP isolates (35.5%) were reported in Egypt during 2019 [36]. This high prevalence further restricts last-line therapy options, potentially leading to deterioration of the clinical situation.

Therefore, we analyzed the frequency of resistance and virulence genes of carbapenem-resistant and ESBL-producing pathogenic *K. pneumoniae* in our community to better understand its dangers. The molecular study used in this study to assess resistance and virulence genes frequency among the selected 49 *K. pneumoniae* isolates revealed that *bla-*_*CTXM*_ (76%), *bla*_*− TEM*_ (63%) were both the most frequent resistance genes, while *bla*_*− KPC*_ was the least common (8%). This could be attributed to the frequent use of cefotaxime and ceftriaxone worldwide [43, 44].

Our results showed that *bla-*_*NDM*_ (51%) showed the highest prevalence among carbapenem-resistant genes, which was strongly associated with phenotypic CRKP (89%), followed by *bla*_*− OXA−48*_ (18%). These findings agree with local studies among outpatients in Egypt during 2023 [45] and other global studies [46, 47]. These data highlight plasmid-mediated dissemination of carbapenemase genes in UTI community patients [47]. Also, the co-occurrence of these genes showed a significant positive relationship.

In our study, phenotypic CRKP showed a significant positive association with *bla-*_*TEM*_ alone and in combination with *bla*_*− CTXM*_ (*P* = 0.049 and 0.038, respectively), consistent with reports linking CRKP to coexisting ESBL genes [47, 48]. This highlights the growing CRKP burden in community UTI cases, a worrying trend given carbapenems are last-line agents. The PDR isolates were positively associated with the *bla*_*− Oxa−48*_ and *bla-*_*KPC*,_ while *bla*_*− Oxa−48*_ showed a significant negative relationship with MDR strains. On the other hand, MDR isolates also had a negative association with coexisting *bla-*_*CTXM*_ and *bla-*_*TEM*_, while XDR isolates were positively linked to *bla*_*− TEM*_ and *bla-*_*NDM*_.

Among ESBL producers, *bla-*_*CTXM*_ was most frequent (87.5%), followed by *bla*_*− TEM*_ (72.5%), aligning with previous Egyptian data, which could be attributed to widespread prescriptions of cephalosporins in Egypt [49, 50]. The co-occurrence of *bla*_*− CTXM*_ and *bla-*_*TEM*_ (67.5%) in our results matched findings in other African communities [51, 52]. Notably, *bla-*_NDM_ was present in 62.5% of ESBL producers in our study, contrasting with higher rates in non-ESBL producers reported among hospitalized patients [53]. Such differences likely reflect variations in antibiotic policies, patient populations, and infection control practices.

As the virulence factors of *K. pneumoniae* seem to be important in designing effective strategies for treating infections caused by these microorganisms, we investigated the virulence profile of the selected *K. pneumoniae* isolates. The results showed that *fim-H* and *mrkD* genes were predominant in nearly all resistant and ESBL-producing isolates (100% in MDR, PDR, and 93% in XDR), underscoring their role in uroepithelial adhesion and biofilm formation. The capsule regulator *rmpA* showed a significantly positive relationship to PDR and CRKP, suggesting enhanced pathogenicity in resistant strains. A study in 2019 proved the positive relationship between the capsule-associated virulence gene *rmpA* and CRKP among urine isolates of Egyptian patients, which agreed with our results [36]. This highlights the increasing rate of CRKP by *rmpA*-positive isolates in Egypt over time. Therefore, controlling the spread of this organism in the general community is a major concern. On the other hand, *rmpA* showed a significantly negative relationship with MDR strains, the same as *uge* showed a significantly negative relationship with PDR. Interestingly, sensitive isolates harbored more virulence genes overall, supporting the concept of a trade-off between resistance and virulence in some strains.

Gender-based differences indicated a higher prevalence of carbapenemase genes (*bla*_*− NDM*_ and *bla-*_*OXA−48*_) and *rmpA* in male patients, potentially reflecting sex-based immune differences and behavioral factors [54, 55].

In our study, a statistical analysis was performed to identify the possible association between antimicrobial resistance genes and virulence gene alterations in *K. pneumoniae* strains. The results showed that *bla-*_*CTXM*_ was in a negative relationship with *bla*_*− KPC*,_ as well as *bla*_*− KPC*_ with *gyr-B-2*. On the other hand, a positive association between the virulence gene *rmpA* and the carbapenemase genes (*bla-*_*NDM*_ and *bla-*_*KPC*_) was detected. Furthermore, there was a significant positive relationship between the co-existence of the two virulent genes *fim-H* and *mrkD*. These findings highlight the function of fimbriae in urinary tract colonization, which is consistent with many other global previous studies [18, 20].

Overall, this study highlights alarmingly high rates of PDR, CRKP, and ESBL-producing *K. pneumoniae* in Egyptian community UTI cases, which underscores the urgent need for antimicrobial stewardship, strict infection control, and genomic surveillance to control the spread of these high-risk clones in the community.

## Limitations

While this study provides valuable data on the resistance and virulence profiles of *K. pneumoniae* in community-acquired UTIs, a few limitations should be noted. The study design was cross-sectional and did not allow for follow-up of clinical outcomes or assessment of treatment responses. Additionally, virulent characters were assessed solely at the genotypic level, without phenotypic confirmation. Despite these limitations, the study offers one of the first detailed analyses combining both phenotypic and genotypic data in non-hospitalized Egyptian patients. It serves as a strong foundation for future research.

## Conclusion

This study highlights the significant levels of antibiotic resistance among *K. pneumoniae* isolates from Egyptian community patients. Genotypic analysis revealed high frequencies of resistance genes along with a widespread distribution of virulence genes. The *rmpA* gene was positively associated with resistance genes (*bla-*_*NDM*_, *bla-*_*KPC*_). Notably, the positive association between virulence and resistance characteristics alarmingly limits treatment options in the community and underscores the urgent need for novel therapeutic strategies.

## Supplementary Information

Below is the link to the electronic supplementary material.

Supplementary Material 1

## Data Availability

Data is provided within the manuscript.

## Abbreviations

UTI: Urinary tract infection
K. Pneumoniae: *Klebsiella pneumoniae*
MIC: Minimum inhibitory concentration
DL/AST: DL Microbial ID/Antimicrobial Susceptibility Testing
ESBL: Extended-spectrum beta-lactamases
PCR: polymerase chain reaction
PDR: Pan drug-resistant
CRKP: Carbapenem-resistant *K. pneumoniae*
UTIs: Urinary tract infections
NDM: New Delhi metallo-β-lactamase
MBL: Metallo-β-lactamase
OXA: oxacillinase
CTX-M-ases: Cefotaximases
LPSs: Lipopolysaccharides
Fim-H: Fimbrial adhesins
uge: uridine diphosphate galacturonate 4-epimerase
CFU: Colony Forming Unit
CDC: Centers for Disease Control and Prevention
DDST: Double Disk Synergy Test
CLSI: Clinical and Laboratory Standards Institute
MH: Muller Hinton
AMC: amoxicillin-clavulanic acid
F: Nitrofurantoin
CZ: Cefazolin
AMP: Ampicillin
CN: Gentamycin
IPM: Imipenem
ERT: Ertapenem
TZP: Tazobactam/piperacillin
SXT: Sulfamethoxazole /Trimethoprim
FEP: Cefepime
CXM: Cefuroxime
CZX: Ceftizoxime
FOX: Cefalexin
LEV: Levofloxacin
SAM: Ampicillin/Sulbactam
AK: Amikacin
MEM: Meropenem
CAZ: Ceftazidime
DNA: Deoxyribonucleic Acid
XDR: Extensively drug-resistant
MDR: Multidrug-resistant
CA-UTIs: Community-acquired urinary tract infections
WHO: World Health Organization
