# Supplementary material for: Prevalence and genotypic–phenotypic association of highly resistant Klebsiella pneumoniae in community-acquired urinary tract infections
Source: BMC Infect Dis. 2025 Nov 22;25:1665. doi: 10.1186/s12879-025-12071-2 (PMC12659173; doi:10.1186/s12879-025-12071-2)
Supplement: Supplementary file 1 — Supplementary Material 1 [file 12879_2025_12071_MOESM1_ESM.docx]

**Table S1. Minimum inhibitory concentration (MIC) values of different antibiotics tested against the tested isolates of *Klebsiella pneumoniae*.**

| **I D** | **f** | **C Z** | **C N** | **AM P** | **I P M** | **E R T** | **T Z P** | **S X T** | **F E P** | **CXM** | **C T X** | **F O X** | **LE V** | **S A M** | **ME M** | **A K** | **C A Z** |
| --- | --- | --- | --- | --- | --- | --- | --- | --- | --- | --- | --- | --- | --- | --- | --- | --- | --- |
| **k 1** | **3 2 (S)** | **≤ 2 (S)** | **≤ 1 (S)** | **≥3 2 (R)** | **≤ 0.2 5 (S)** | **≤0.015 (S)** | **≤ 4 / 4 (S)** | **≤0.5/9.5 (S)** | **≤ 0.1 2 (S)** | **≤4 (S)** | **≤ 0.1 2 (S)** | **≤ 8 (S)** | **≤0.06 (S)** | **≤ 8 / 4 (S)** | **≤0.06 (S)** | **≤ 4 (S)** | **≤ 0.5 (S)** |
| **k 2** | **3 2 (S)** | **≥ 32 (R)** | **≤ 1 (S)** | **≥3 2 (R)** | **≤0.2 5 (S)** | **≤0.015 (S)** | **≤ 4 / 4 (S)** | **≥8/152 (R)** | **2 (S)** | **8 (S)** | **1 (S)** | **≤ 8 (S)** | **≤0.06 (S)** | **≤ 8 / 4 (S)** | **≤0.06 (S)** | **≤4 (S)** | **4 (S)** |
| **k 3** | **≥128 (R)** | **≤ 2 (S)** | **≤ 1 (S)** | **≥3 2 (R)** | **≤ 0.2 5 (S)** | **≤0.015 (S)** | **≤4 / 4 (S)** | **≥8/152 (R)** | **≤ 0.1 2 (S)** | **≤ 4 (S)** | **≤ 0.1 2 (S)** | **≤ 8 (S)** | **≥ 8 (R)** | **2 (R)** | **≤0.06 (S)** | **≤4 (S)** | **≤ 0.5 (S)** |
| **k 4** | **6 4 (R)** | **≥ 3 2 (R)** | **≤1 (S)** | **≥3 2 (R)** | **≤ 0.2 5 (S)** | **≤0.015 (S)** | **≤ 4 / 4 (S)** | **≤0.5/9.5 (S)** | **4 (R)** | **≥64 (R)** | **≥ 6 4 (R)** | **≤ 8 (S)** | **0. 1 2 (S)** | **2 (R)** | **2 (R)** | **≤ 4 (S)** | **8 (R)** |
| **k 5** | **≥128 (R)** | **≥ 3 2 (R)** | **≥16 (R)** | **≥3 2 (R)** | **4 (R)** | **8 (R)** | **≥128/4 (R)** | **≥8/152 (R)** | **≥ 3 2 (R)** | **≥64 (R)** | **≥ 6 4 (R)** | **≥64 (R)** | **≥ 8 (R)** | **≥32/16 (R)** | **8 (R)** | **≥64 (R)** | **≥ 3 2 (R)** |
| **k 6** | **≤16 (S)** | **≥ 3 2 (R)** | **≥16 (R)** | **≥3 2 (R)** | **1 (S)** | **4 (R)** | **≥128/4 (R)** | **≥8/152 (R)** | **≥ 3 2 (R)** | **≥64 (R)** | **≥ 6 4 (R)** | **≥64 (R)** | **1 (S)** | **≥32/16 (R)** | **2 (R)** | **≥64 (R)** | **≥ 3 2 (R)** |
| **k 7** | **≤16 (S)** | **≥ 3 2 (R)** | **≤ 1 (S)** | **≥3 2 (R)** | **≤ 0.2 5 (S)** | **0.5 (S)** | **≤ 4 / 4 (S)** | **≥8/152 (R)** | **8 (R)** | **≤64 (S)** | **≥ 6 4 (R)** | **≥64 (R)** | **0. 1 2 (S)** | **≥32/16 (R)** | **1 (S)** | **≤ 4 (S)** | **≥ 3 2 (R)** |
| **k 8** | **3 2 (S)** | **≤ 2 (S)** | **8 (R)** | **≥3 2 (R)** | **≤0.2 5 (S)** | **≤0.015 (S)** | **≤ 4 / 4 (S)** | **≥8/152 (R)** | **≤ 0.1 2 (S)** | **≤ 4 (S)** | **≤ 0.1 2 (S)** | **≤ 8 (S)** | **≥ 8 (R)** | **≤ 8 / 4 (S)** | **≤0.06 (S)** | **≤ 4 (S)** | **≤ 0.5 (S)** |
| **k 9** | **6 4 (R)** | **≥ 3 2 (R)** | **≤1 (S)** | **≥3 2 (R)** | **≤ 0.2 5 (S)** | **0.5 (S)** | **4 (S)** | **≥8/152 (R)** | **8 (R)** | **≥64 (R)** | **≥ 6 4 (R)** | **≤ 8 (S)** | **≥ 8 (R)** | **≥32/16 (R)** | **≤0.06 (S)** | **≤4 (S)** | **≥ 3 2 (R)** |
| **k1 0** | **3 2 (S)** | **≥ 3 2 (R)** | **≤ 1 (S)** | **≥3 2 (R)** | **≤ 0.2 5 (S)** | **0.5 (S)** | **4 (S)** | **≥8/152 (R)** | **8 (R)** | **≥64 (R)** | **≥ 6 4 (R)** | **≤ 8 (S)** | **≥ 8 (R)** | **≥32/16 (R)** | **≤0.06 (S)** | **≤ 4 (S)** | **≥ 3 2 (R)** |
| **k1 1** | **3 2 (S)** | **≥ 3 2 (R)** | **≤ 1 (S)** | **≥3 2 (R)** | **≤ 0.2 5 (S)** | **0.5 (S)** | **≤ 4 / 4 (S)** | **≥8/152 (R)** | **4 (R)** | **≥64 (R)** | **≥ 6 4 (R)** | **≤ 8 (S)** | **0. 1 2 (S)** | **2 (R)** | **≤0.06 (S)** | **≤ 4 (S)** | **1 6 (R)** |
| **k1 2** | **≤16 (S)** | **≥ 3 2 (R)** | **8 (R)** | **≥3 2 (R)** | **≤ 0.2 5 (S)** | **0.5 (S)** | **≤ 4 / 4 (S)** | **≥8/152 (R)** | **8 (R)** | **≥64 (R)** | **≥ 6 4 (R)** | **≤ 8 (S)** | **0. 1 2 (S)** | **≥32/16 (R)** | **≤0.06 (S)** | **≤ 4 (S)** | **8 (R)** |
| **k1 3** | **6 4 (R)** | **≤ 2 (S)** | **≤1 (S)** | **≥3 2 (R)** | **≤ 0.2 5 (S)** | **≤0.015 (S)** | **≤ 4 / 4 (S)** | **≤0.5/9.5 (S)** | **≤ 0.1 2 (S)** | **≤ 4 (S)** | **≤0.1 2 (S)** | **≤ 8 (S)** | **≤0.06 (S)** | **≤ 8 / 4 (S)** | **≤0.06 (S)** | **≤4 (S)** | **≤ 0.5 (S)** |
| **k1 4** | **6 4 (R)** | **≥ 3 2 (R)** | **≤1 (S)** | **≥3 2 (R)** | **≤ 0.2 5 (S)** | **0.5 (S)** | **≤ 4 / 4 (S)** | **≤0.5/9.5 (S)** | **8 (R)** | **≥64 (R)** | **≥ 6 4 (R)** | **≤ 8 (S)** | **≥ 8 (R)** | **≥32/16 (R)** | **≤0.06 (S)** | **≤ 4 (S)** | **1 6 (R)** |
| **k1 5** | **≥128 (R)** | **≥ 3 2 (R)** | **≥16 (R)** | **≥3 2 (R)** | **4 (R)** | **8 (R)** | **≥128/4 (R)** | **≥8/152 (R)** | **≥ 3 2 (R)** | **≥64 (R)** | **≥ 6 4 (R)** | **≥64 (R)** | **≥ 8 (R)** | **≥32/16 (R)** | **8 (R)** | **≥64 (R)** | **≥ 3 2 (R)** |
| **k1 6** | **≥128 (R)** | **≥ 3 2 (R)** | **≥16 (R)** | **≥3 2 (R)** | **4 (R)** | **8 (R)** | **≥128/4 (R)** | **≥8/152 (R)** | **≥ 3 2 (R)** | **≥64 (R)** | **≥ 6 4 (R)** | **≥64 (R)** | **≥ 8 (R)** | **≥32/16 (R)** | **8 (R)** | **≥64 (R)** | **≥ 3 2 (R)** |
| **k1 7** | **≥128 (R)** | **≥ 3 2 (R)** | **≤1 (S)** | **≥3 2 (R)** | **16 (R)** | **≥ 1 6 (R)** | **≥128/4 (R)** | **0.05 (S)** | **≥ 3 2 (R)** | **≥64 (R)** | **≥ 6 4 (R)** | **≥64 (R)** | **≥ 8 (R)** | **≥32/16 (R)** | **≥ 3 2 (R)** | **3 2 (R)** | **≥ 3 2 (R)** |
| **k1 8** | **≥128 (R)** | **≥ 3 2 (R)** | **≥16 (R)** | **≥3 2 (R)** | **16 (R)** | **≥ 1 6 (R)** | **≥128/4 (R)** | **≥8/152 (R)** | **≥ 3 2 (R)** | **≥64 (R)** | **≥ 6 4 (R)** | **≥64 (R)** | **≥ 8 (R)** | **≥32/16 (R)** | **≥ 3 2 (R)** | **≥64 (R)** | **≥ 3 2 (R)** |
| **k1 9** | **≥128 (R)** | **≥ 3 2 (R)** | **≥16 (R)** | **≥3 2 (R)** | **16 (R)** | **≥ 1 6 (R)** | **≥128/4 (R)** | **≥8/152 (R)** | **≥ 3 2 (R)** | **≥64 (R)** | **≥ 6 4 (R)** | **≥64 (R)** | **≥ 8 (R)** | **≥32/16 (R)** | **≥ 3 2 (R)** | **≥64 (R)** | **≥ 3 2 (R)** |
| **k2 0** | **≥128 (R)** | **≥ 3 2 (R)** | **≤ 1 (S)** | **≥3 2 (R)** | **8 (R)** | **≥ 1 6 (R)** | **≥128/4 (R)** | **≥8/152 (R)** | **≥ 3 2 (R)** | **≥64 (R)** | **≥ 6 4 (R)** | **≥64 (R)** | **≥ 8 (R)** | **≥32/16 (R)** | **≥ 3 2 (R)** | **≤ 4 (S)** | **≥ 3 2 (R)** |
| **k2 1** | **≥128 (R)** | **≥ 3 2 (R)** | **≥16 (R)** | **≥3 2 (R)** | **16 (R)** | **≥ 1 6 (R)** | **≥128/4 (R)** | **≥8/152 (R)** | **≥ 3 2 (R)** | **≥64 (R)** | **≥ 6 4 (R)** | **≥64 (R)** | **≥ 8 (R)** | **≥32/16 (R)** | **≥ 3 2 (R)** | **≥64 (R)** | **≥ 3 2 (R)** |
| **k2 2** | **≥128 (R)** | **≥ 3 2 (R)** | **≥16 (R)** | **≥3 2 (R)** | **16 (R)** | **≥ 1 6 (R)** | **≥128/4 (R)** | **≥8/152 (R)** | **≥ 3 2 (R)** | **≥64 (R)** | **≥ 6 4 (R)** | **≥64 (R)** | **≥ 8 (R)** | **≥32/16 (R)** | **≥ 3 2 (R)** | **≥64 (R)** | **≥ 3 2 (R)** |
| **k2 3** | **≥128 (R)** | **≥ 3 2 (R)** | **≥16 (R)** | **≥3 2 (R)** | **≥ 3 2 (R)** | **≥ 1 6 (R)** | **≥128/4 (R)** | **≥8/152 (R)** | **≥ 3 2 (R)** | **≥64 (R)** | **≥ 6 4 (R)** | **≥64 (R)** | **≥ 8 (R)** | **≥32/16 (R)** | **≥ 3 2 (R)** | **≥64 (R)** | **≥ 32 (R)** |
| **k2 4** | **≥128 (R)** | **≥ 3 2 (R)** | **≥16 (R)** | **≥3 2 (R)** | **4 (R)** | **8 (R)** | **≥128/4 (R)** | **≥8/152 (R)** | **≥ 3 2 (R)** | **≥64 (R)** | **≥ 6 4 (R)** | **≥64 (R)** | **≥ 8 (R)** | **≥32/16 (R)** | **8 (R)** | **≥64 (R)** | **≥ 32 (R)** |
| **k2 5** | **≥128 (R)** | **≥ 3 2 (R)** | **≥16 (R)** | **≥3 2 (R)** | **16 (R)** | **≥ 1 6 (R)** | **≥128/4 (R)** | **≥8/152 (R)** | **≥ 3 2 (R)** | **≥64 (R)** | **≥ 6 4 (R)** | **≥64 (R)** | **≥ 8 (R)** | **≥32/16 (R)** | **≥ 3 2 (R)** | **≥64 (R)** | **≥ 32 (R)** |
| **k2 6** | **6 4 (R)** | **≥ 3 2 (R)** | **8 (R)** | **≥3 2 (R)** | **1 (S)** | **0.5 (S)** | **≤ 4 / 4 (S)** | **≥8/152 (R)** | **8 (R)** | **≥64 (R)** | **≥ 6 4 (R)** | **≤ 8 (S)** | **≥ 8 (R)** | **≥32/16 (R)** | **≤0.06 (S)** | **≤ 4 (S)** | **16 (R)** |
| **k2 7** | **≤16 (S)** | **≥ 3 2 (R)** | **≤ 1 (S)** | **≥3 2 (R)** | **≤ 0.2 5 (S)** | **≤0.015 (S)** | **≤4 / 4 (S)** | **≥8/152 (R)** | **4 (R)** | **≥64 (R)** | **32 (R)** | **≤ 8 (S)** | **0. 1 2 (S)** | **≤ 8 / 4 (S)** | **≤0.06 (S)** | **≤ 4 (S)** | **16 (R)** |
| **k2 8** | **3 2 (S)** | **8 (S)** | **≤ 1 (S)** | **≥3 2 (R)** | **≤ 0.2 5 (S)** | **≤0.015 (S)** | **≤ 4 / 4 (S)** | **≤0.5/9.5 (S)** | **≤ 0.1 2 (S)** | **≤ 4 (S)** | **≤ 0.1 2 (S)** | **≤ 8 (S)** | **≤0.06 (S)** | **≤ 8 / 4 (S)** | **≤0.06 (S)** | **≤ 4 (S)** | **≤ 0.5 (S)** |
| **k2 9** | **≥128 (R)** | **≥ 3 2 (R)** | **8 (R)** | **≥3 2 (R)** | **≤ 0.2 5 (S)** | **0.5 (S)** | **≤ 4 / 4 (S)** | **≥8/152 (R)** | **≥ 32 (R)** | **≥64 (R)** | **≥ 6 4 (R)** | **≤ 8 (S)** | **4 (R)** | **≥32/16 (R)** | **≤0.06 (S)** | **≤ 4 (S)** | **≥ 3 2 (R)** |
| **k3 0** | **≥128 (R)** | **≥ 3 2 (R)** | **≥16 (R)** | **≥3 2 (R)** | **8 (R)** | **≥ 1 6 (R)** | **≥128/4 (R)** | **≥8/152 (R)** | **≥ 3 2 (R)** | **≥64 (R)** | **≥ 6 4 (R)** | **≥64 (R)** | **≥ 8 (R)** | **≥32/16 (R)** | **≥ 3 2 (R)** | **≥64 (R)** | **≥ 3 2 (R)** |
| **k3 1** | **≥128 (R)** | **≥ 3 2 (R)** | **≥16 (R)** | **≥3 2 (R)** | **16 (R)** | **≥ 1 6 (R)** | **≥128/4 (R)** | **≥8/152 (R)** | **≥ 3 2 (R)** | **≥64 (R)** | **≥ 6 4 (R)** | **≥64 (R)** | **≥ 8 (R)** | **≥32/16 (R)** | **≥ 3 2 (R)** | **≥64 (R)** | **≥ 3 2 (R)** |
| **k3 2** | **3 2 (S)** | **≥ 3 2 (R)** | **≥16 (R)** | **≥3 2 (R)** | **2 (R)** | **8 (R)** | **≥128/4 (R)** | **≥8/152 (R)** | **≥ 3 2 (R)** | **≥64 (R)** | **≥ 6 4 (R)** | **≥64 (R)** | **2 (S)** | **≥32/16 (R)** | **4 (R)** | **≥64 (R)** | **≥ 3 2 (R)** |
| **k3 3** | **3 2 (S)** | **≥ 3 2 (R)** | **≤ 1 (S)** | **≥3 2 (R)** | **1 (S)** | **0.5 (S)** | **≤ 4 / 4 (S)** | **≥8/152 (R)** | **8 (R)** | **≥64 (R)** | **≥ 6 4 (R)** | **≤ 8 (S)** | **0. 1 2 (S)** | **2 (R)** | **1 (S)** | **≤ 4 (S)** | **16 (R)** |
| **k3 4** | **3 2 (S)** | **≥ 3 2 (R)** | **≥16 (R)** | **≥3 2 (R)** | **4 (R)** | **8 (R)** | **≥128/4 (R)** | **≥8/152 (R)** | **≥ 3 2 (R)** | **≥64 (R)** | **≥ 6 4 (R)** | **≥64 (R)** | **1 (S)** | **≥32/16 (R)** | **4 (R)** | **≥64 (R)** | **≥ 3 2 (R)** |
| **k3 5** | **≥128 (R)** | **≥ 3 2 (R)** | **≥16 (R)** | **≥3 2 (R)** | **16 (R)** | **≥ 1 6 (R)** | **≥128/4 (R)** | **≥8/152 (R)** | **≥ 3 2 (R)** | **≥64 (R)** | **≥ 6 4 (R)** | **≥64 (R)** | **≥ 8 (R)** | **≥32/16 (R)** | **≥ 3 2 (R)** | **≥64 (R)** | **≥ 3 2 (R)** |
| **k3 6** | **3 2 (S)** | **≥ 3 2 (R)** | **≤ 1 (S)** | **≥3 2 (R)** | **≤ 0.2 5 (S)** | **0.5 (S)** | **≤ 4 / 4 (S)** | **≥8/152 (R)** | **8 (R)** | **≥64 (R)** | **≥ 6 4 (R)** | **≤ 8 (S)** | **≤0.06 (S)** | **≤ 8 / 4 (S)** | **≤0.06 (S)** | **≤ 4 (S)** | **16 (R)** |
| **k3 7** | **3 2 (S)** | **≤ 2 (S)** | **≤1 (S)** | **≥3 2 (R)** | **≤ 0.2 5 (S)** | **≤0.015 (S)** | **≤ 4 / 4 (S)** | **≤0.5/9.5 (S)** | **≤ 0.1 2 (S)** | **≤ 4 (S)** | **≤ 0.1 2 (S)** | **≤ 8 (S)** | **≤0.06 (S)** | **≤ 8 / 4 (S)** | **≤0.06 (S)** | **≤ 4 (S)** | **≤ 0.5 (S)** |
| **k3 8** | **≤16 (S)** | **≥ 3 2 (R)** | **≥16 (R)** | **≥3 2 (R)** | **1 (S)** | **4 (R)** | **≥128/4 (R)** | **≥8/152 (R)** | **≥ 3 2 (R)** | **≥64 (R)** | **≥ 6 4 (R)** | **≥64 (R)** | **2 (S)** | **≥32/16 (R)** | **2 (R)** | **≥64 (R)** | **≥ 3 2 (R)** |
| **K39** | **≥128 (R)** | **≥ 3 2 (R)** | **≥16 (R)** | **≥3 2 (R)** | **4 (R)** | **8 (R)** | **≥128/4 (R)** | **≥8/152 (R)** | **≥ 3 2 (R)** | **≥64 (R)** | **≥ 6 4 (R)** | **≥64 (R)** | **≥ 8 (R)** | **≥32/16 (R)** | **8 (R)** | **≤ 4 (S)** | **≥ 3 2 (R)** |
| **k4 0** | **≥128 (R)** | **≥ 3 2 (R)** | **≥16 (R)** | **≥3 2 (R)** | **≥ 3 2 (R)** | **≥ 1 6 (R)** | **≥128/4 (R)** | **≥8/152 (R)** | **≥ 3 2 (R)** | **≥64 (R)** | **≥ 6 4 (R)** | **≥64 (R)** | **≥ 8 (R)** | **≥32/16 (R)** | **≥ 3 2 (R)** | **≥64 (R)** | **≥ 3 2 (R)** |
| **k4 1** | **≥128 (R)** | **≥ 3 2 (R)** | **≥16 (R)** | **≥3 2 (R)** | **≥ 3 2 (R)** | **≥ 1 6 (R)** | **≥128/4 (R)** | **≥8/152 (R)** | **≥ 3 2 (R)** | **≥64 (R)** | **≥ 6 4 (R)** | **≥64 (R)** | **≥ 8 (R)** | **≥32/16 (R)** | **≥ 3 2 (R)** | **≥64 (R)** | **≥ 3 2 (R)** |
| **k4 2** | **3 2 (S)** | **≥ 3 2 (R)** | **≥16 (R)** | **≥3 2 (R)** | **1 (S)** | **0.5 (S)** | **≤4 / 4 (S)** | **≥8/152 (R)** | **8 (R)** | **≥64 (R)** | **≥ 6 4 (R)** | **≤ 8 (S)** | **0. 1 2 (S)** | **2 (R)** | **1 (S)** | **≤ 4 (S)** | **16 (R)** |
| **k4 3** | **≥128 (R)** | **≥ 3 2 (R)** | **≥16 (R)** | **≥3 2 (R)** | **≥ 3 2 (R)** | **≥ 1 6 (R)** | **≥128/4 (R)** | **≥8/152 (R)** | **≥ 3 2 (R)** | **≥64 (R)** | **≥ 6 4 (R)** | **≥64 (R)** | **≥ 8 (R)** | **≥32/16 (R)** | **≥ 3 2 (R)** | **≥64 (R)** | **≥ 3 2 (R)** |
| **k4 4** | **3 2 (S)** | **≥ 3 2 (R)** | **8 (R)** | **≥3 2 (R)** | **4 (R)** | **≥ 1 6 (R)** | **≥128/4 (R)** | **≥8/152 (R)** | **≥ 3 2 (R)** | **≥64 (R)** | **≥ 6 4 (R)** | **≥64 (R)** | **2 (S)** | **≥32/16 (R)** | **8 (R)** | **≤ 4 (S)** | **≥ 3 2 (R)** |
| **k4 5** | **3 2 (S)** | **≥ 3 2 (R)** | **≤ 1 (S)** | **≥3 2 (R)** | **≤ 0.2 5 (S)** | **≤0.015 (S)** | **≤ 4 / 4 (S)** | **≥8/152 (R)** | **4 (R)** | **≥64 (R)** | **≥ 6 4 (R)** | **≤8 (S)** | **0. 1 2 (S)** | **≤ 8 / 4 (S)** | **≤0.06 (S)** | **≤ 4 (S)** | **16 (R)** |
| **k4 6** | **≥128 (R)** | **≥ 3 2 (R)** | **≤ 1 (S)** | **≥3 2 (R)** | **≤ 0.2 5 (S)** | **2 (R)** | **8 (R)** | **≤0.5/9.5 (S)** | **≥ 3 2 (R)** | **≥64 (R)** | **≥ 6 4 (R)** | **32 (R)** | **≥ 8 (R)** | **≥32/16 (R)** | **1 (S)** | **≤ 4 (S)** | **≥ 3 2 (R)** |
| **k4 7** | **≥128 (R)** | **≥ 3 2 (R)** | **≤ 1 (S)** | **≥3 2 (R)** | **16 (R)** | **≥ 1 6 (R)** | **≥128/4 (R)** | **≤0.5/9.5 (S)** | **≥ 3 2 (R)** | **≥64 (R)** | **≥ 6 4 (R)** | **≥64 (R)** | **≥ 8 (R)** | **≥32/16 (R)** | **≥ 3 2 (R)** | **≤ 4 (S)** | **≥ 3 2 (R)** |
| **k4 8** | **3 2 (S)** | **≥ 3 2 (R)** | **≥16 (R)** | **≥3 2 (R)** | **2 (R)** | **4 (R)** | **≥128/4 (R)** | **≥8/152 (R)** | **≥ 3 2 (R)** | **≥64 (R)** | **≥ 6 4 (R)** | **≥64 (R)** | **2 (S)** | **≥32/16 (R)** | **2 (R)** | **≥64 (R)** | **≥ 3 2 (R)** |
| **K49** | **3 2 (S)** | **≥ 3 2 (R)** | **≤ 1 (S)** | **≥3 2 (R)** | **≤ 0.2 5 (S)** | **0.5 (S)** | **≤4 / 4 (S)** | **≥8/152 (R)** | **4 (R)** | **≥64 (R)** | **32 (R)** | **≤ 8 (S)** | **0. 1 2 (S)** | **≤ 8 / 4 (S)** | **≤0.06 (S)** | **≤ 4 (S)** | **4 (S)** |
| **k5 0** | **3 2 (S)** | **≥ 3 2 (R)** | **≥16 (R)** | **≥3 2 (R)** | **4 (R)** | **8 (R)** | **≥128/4 (R)** | **≥8/152 (R)** | **≥ 3 2 (R)** | **≥64 (R)** | **≥ 6 4 (R)** | **≥64 (R)** | **2 (S)** | **≥32/16 (R)** | **8 (R)** | **≤ 4 (S)** | **≥ 3 2 (R)** |
| **k5 1** | **≥128 (R)** | **≥ 3 2 (R)** | **≥16 (R)** | **≥3 2 (R)** | **≥ 3 2 (R)** | **≥ 1 6 (R)** | **≥128/4 (R)** | **≥8/152 (R)** | **≥ 3 2 (R)** | **≥64 (R)** | **≥ 6 4 (R)** | **≥64 (R)** | **≥ 8 (R)** | **≥32/16 (R)** | **≥ 3 2 (R)** | **≥64 (R)** | **≥ 3 2 (R)** |
| **k5 2** | **3 2 (S)** | **≥ 3 2 (R)** | **8 (R)** | **≥3 2 (R)** | **4 (R)** | **≥ 1 6 (R)** | **≥128/4 (R)** | **≥8/152 (R)** | **≥ 3 2 (R)** | **≥64 (R)** | **≥ 6 4 (R)** | **≥64 (R)** | **2 (S)** | **≥32/16 (R)** | **8 (R)** | **≤ 4 (S)** | **≥ 3 2 (R)** |
| **k5 3** | **≥128 (R)** | **≥ 3 2 (R)** | **≥16 (R)** | **≥3 2 (R)** | **16 (R)** | **≥ 1 6 (R)** | **≥128/4 (R)** | **≥8/152 (R)** | **≥ 3 2 (R)** | **≥64 (R)** | **≥ 6 4 (R)** | **≥64 (R)** | **≥ 8 (R)** | **≥32/16 (R)** | **≥ 3 2 (R)** | **≥64 (R)** | **≥ 3 2 (R)** |
| **k5 4** | **≤16 (S)** | **≥ 3 2 (R)** | **≤ 1 (S)** | **≥3 2 (R)** | **≤ 0.2 5 (S)** | **≤0.015 (S)** | **≤ 4 / 4 (S)** | **≤0.5/9.5 (S)** | **≥ 3 2 (R)** | **≥64 (R)** | **≥ 6 4 (R)** | **≥64 (R)** | **≤0.06 (S)** | **≤ 8 / 4 (S)** | **≤0.06 (S)** | **≤ 4 (S)** | **≥ 3 2 (R)** |
| **k5 5** | **≥128 (R)** | **≥ 3 2 (R)** | **≤ 1 (S)** | **≥3 2 (R)** | **≤0.2 5 (S)** | **≤0.015 (S)** | **>128/4 (R)** | **≥8/152 (R)** | **≥ 3 2 (R)** | **≥64 (R)** | **≥ 6 4 (R)** | **≥64 (R)** | **≥ 8 (R)** | **≥32/16 (R)** | **≤0.06 (S)** | **≤ 4 (S)** | **≥ 3 2 (R)** |
| **k5 6** | **≥128 (R)** | **≥ 3 2 (R)** | **≥16 (R)** | **≥3 2 (R)** | **≥ 3 2 (R)** | **≥ 1 6 (R)** | **≥128/4 (R)** | **≥8/152 (R)** | **≥ 3 2 (R)** | **≥64 (R)** | **≥ 6 4 (R)** | **≥64 (R)** | **≥ 8 (R)** | **≥32/16 (R)** | **≥ 3 2 (R)** | **≥64 (R)** | **≥ 3 2 (R)** |
| **k5 7** | **≥128 (R)** | **≥ 3 2 (R)** | **≤ 1 (S)** | **≥3 2 (R)** | **≤ 0.2 5 (S)** | **≤0.015 (S)** | **≥128/4 (R)** | **≥8/152 (R)** | **≥ 3 2 (R)** | **≥64 (R)** | **≥ 6 4 (R)** | **≥64 (R)** | **≥ 8 (R)** | **2 (R)** | **≤0.06 (S)** | **≤ 4 (S)** | **≥ 3 2 (R)** |
| **k5 8** | **6 4 (R)** | **≥ 3 2 (R)** | **≤ 1 (S)** | **≥3 2 (R)** | **≤ 0.2 5 (S)** | **0.5 (S)** | **≥128/4 (R)** | **≥8/152 (R)** | **≥ 3 2 (R)** | **≥64 (R)** | **≥ 6 4 (R)** | **≥64 (R)** | **≥ 8 (R)** | **≤ 8 / 4 (S)** | **≤0.06 (S)** | **≤ 4 (S)** | **≥ 3 2 (R)** |
| **k5 9** | **≤16 (S)** | **≥ 3 2 (R)** | **≤ 1 (S)** | **≥3 2 (R)** | **≤0.2 5 (S)** | **≤0.015 (S)** | **≤ 4 / 4 (S)** | **≥8/152 (R)** | **≥ 3 2 (R)** | **≥64 (R)** | **≥ 6 4 (R)** | **≤ 8 (S)** | **≤0.06 (S)** | **≤ 8 / 4 (S)** | **≤0.06 (S)** | **≤ 4 (S)** | **≥ 3 2 (R)** |
| **k6 0** | **≤16 (S)** | **≥ 3 2 (R)** | **≤ 1 (S)** | **≥3 2 (R)** | **≤ 0.2 5 (S)** | **≤0.015 (S)** | **≤ 4 / 4 (S)** | **≤0.5/9.5 (S)** | **≥ 3 2 (R)** | **≥64 (R)** | **≥ 6 4 (R)** | **≥64 (R)** | **≤0.06 (S)** | **≤8 / 4 (S)** | **≤0.06 (S)** | **≤ 4 (S)** | **≥ 3 2 (R)** |
| **k6 1** | **≤16 (S)** | **≥ 3 2 (R)** | **≤ 1 (S)** | **≥3 2 (R)** | **≤ 0.2 5 (S)** | **≤0.015 (S)** | **≤4 / 4 (S)** | **≥8/152 (R)** | **≥ 3 2 (R)** | **≥64 (R)** | **≥ 6 4 (R)** | **≥64 (R)** | **≤0.06 (S)** | **≤ 8 / 4 (S)** | **≤0.06 (S)** | **≤ 4 (S)** | **≥ 3 2 (R)** |
| **k6 2** | **≤16 (S)** | **≥ 3 2 (R)** | **≤ 1 (S)** | **≥3 2 (R)** | **≤0.2 5 (S)** | **0.5 (S)** | **≤ 4 / 4 (S)** | **≥8/152 (R)** | **≥ 3 2 (R)** | **≥64 (R)** | **≥ 6 4 (R)** | **≤ 8 (S)** | **≤0.06 (S)** | **≤ 8 / 4 (S)** | **≤0.06 (S)** | **≤4 (S)** | **≥ 3 2 (R)** |
| **k6 3** | **3 2 (S)** | **≤2 (S)** | **≤ 1 (S)** | **≤ 8 (S)** | **≤ 0.2 5 (S)** | **≤0.015 (S)** | **≤ 4 / 4 (S)** | **≤0.5/9.5 (S)** | **2 (S)** | **8 (S)** | **1 (S)** | **≤ 8 (S)** | **≤0.06 (S)** | **≤8 / 4 (S)** | **≤0.06 (S)** | **≤ 4 (S)** | **4 (S)** |
| **k6 4** | **3 2 (S)** | **≤ 2 (S)** | **≤ 1 (S)** | **≤ 8 (S)** | **≤ 0.2 5 (S)** | **0.5 (S)** | **≤ 4 / 4 (S)** | **≥8/152 (R)** | **≤ 0.1 2 (S)** | **≤ 4 (S)** | **≤ 0.1 2 (S)** | **≤ 8 (S)** | **≤0.06 (S)** | **≤ 8 / 4 (S)** | **≤0.06 (S)** | **≤ 4 (S)** | **≤ 0.5 (S)** |
| **k6 5** | **3 2 (S)** | **≤ 2 (S)** | **≤ 1 (S)** | **≤ 8 (S)** | **≤ 0.2 5 (S)** | **0.5 (S)** | **≤ 4 / 4 (S)** | **≤0.5/9.5 (S)** | **≤ 0.1 2 (S)** | **≤ 4 (S)** | **≤ 0.1 2 (S)** | **≤ 8 (S)** | **≤0.06 (S)** | **≤ 8 / 4 (S)** | **≤0.06 (S)** | **≤ 4 (S)** | **≤ 0.5 (S)** |
| **k6 6** | **≤16 (S)** | **≥ 3 2 (R)** | **≤ 1 (S)** | **≥3 2 (R)** | **≤ 0.2 5 (S)** | **≤0.015 (S)** | **≤ 4 / 4 (S)** | **≥8/152 (R)** | **≥ 3 2 (R)** | **≥64 (R)** | **≥ 6 4 (R)** | **≥64 (R)** | **≥ 8 (R)** | **≤ 8 / 4 (S)** | **≤0.06 (S)** | **≤ 4 (S)** | **≥ 3 2 (R)** |
| **k6 7** | **3 2 (S)** | **≤ 2 (S)** | **≤ 1 (S)** | **≤ 8 (S)** | **≤ 0.2 5 (S)** | **≤0.015 (S)** | **≤ 4 / 4 (S)** | **≥8/152 (R)** | **≤ 0.1 2 (S)** | **≤ 4 (S)** | **≤ 0.1 2 (S)** | **≤ 8 (S)** | **2 (S)** | **≤ 8 / 4 (S)** | **≤0.06 (S)** | **≤ 4 (S)** | **≤ 0.5 (S)** |
| **k6 8** | **6 4 (R)** | **≥ 3 2 (R)** | **≤ 1 (S)** | **≥3 2 (R)** | **≤ 0.2 5 (S)** | **0.5 (S)** | **≥128/4 (R)** | **≥8/152 (R)** | **≥ 3 2 (R)** | **≥64 (R)** | **≥ 6 4 (R)** | **≥64 (R)** | **2 (S)** | **≥32/16 (R)** | **≤0.06 (S)** | **≤ 4 (S)** | **≥ 3 2 (R)** |
| **k6 9** | **≤16 (S)** | **≥ 3 2 (R)** | **≤ 1 (S)** | **≥3 2 (R)** | **≤ 0.2 5 (S)** | **≤0.015 (S)** | **≤ 4 / 4 (S)** | **≥8/152 (R)** | **≥ 3 2 (R)** | **≥64 (R)** | **≥ 6 4 (R)** | **≤ 8 (S)** | **≥ 8 (R)** | **≤ 8 / 4 (S)** | **≤0.06 (S)** | **≤ 4 (S)** | **≥ 3 2 (R)** |
| **k7 0** | **≤16 (S)** | **≥ 3 2 (R)** | **≤ 1 (S)** | **≥3 2 (R)** | **≤ 0.2 5 (S)** | **≤0.015 (S)** | **≤ 4 / 4 (S)** | **≥8/152 (R)** | **≥ 3 2 (R)** | **≥64 (R)** | **≥ 6 4 (R)** | **≥64 (R)** | **≥ 8 (R)** | **≤ 8 / 4 (S)** | **≤0.06 (S)** | **≤ 4 (S)** | **≥ 3 2 (R)** |
| **k7 1** | **≤16 (S)** | **≥ 3 2 (R)** | **≤ 1 (S)** | **≥3 2 (R)** | **≤ 0.2 5 (S)** | **≤0.015 (S)** | **≥128/4 (R)** | **≥8/152 (R)** | **≥ 3 2 (R)** | **≥64 (R)** | **≥ 6 4 (R)** | **≥64 (R)** | **≥ 8 (R)** | **≥32/16 (R)** | **≤0.06 (S)** | **≤ 4 (S)** | **≥ 3 2 (R)** |
| **k7 2** | **≤16 (S)** | **≥ 3 2 (R)** | **≤ 1 (S)** | **≥3 2 (R)** | **≤ 0.2 5 (S)** | **≤0.015 (S)** | **≥128/4 (R)** | **≥8/152 (R)** | **≥ 3 2 (R)** | **≥64 (R)** | **≥ 6 4 (R)** | **≥64 (R)** | **2 (S)** | **≥32/16 (R)** | **≤0.06 (S)** | **≤ 4 (S)** | **≥ 3 2 (R)** |
| **k7 3** | **3 2 (S)** | **≤ 2 (S)** | **≤ 1 (S)** | **≤ 8 (S)** | **≤ 0.2 5 (S)** | **≤0.015 (S)** | **≤ 4 / 4 (S)** | **≤0.5/9.5 (S)** | **≤ 0.1 2 (S)** | **≤ 4 (S)** | **≤ 0.1 2 (S)** | **≤ 8 (S)** | **≤0.06 (S)** | **≤ 8 / 4 (S)** | **≤0.06 (S)** | **≤ 4 (S)** | **≤ 0.5 (S)** |
| **k7 4** | **≥128 (R)** | **≥ 3 2 (R)** | **≤ 1 (S)** | **≥3 2 (R)** | **≤ 0.2 5 (S)** | **≤0.015 (S)** | **≥128/4 (R)** | **≥8/152 (R)** | **≥ 3 2 (R)** | **≥64 (R)** | **≥ 6 4 (R)** | **≤ 8 (S)** | **2 (S)** | **≥32/16 (R)** | **≤0.06 (S)** | **≤ 4 (S)** | **≥ 3 2 (R)** |
| **k7 5** | **≤16 (S)** | **≥ 3 2 (R)** | **≤ 1 (S)** | **≥3 2 (R)** | **≤ 0.2 5 (S)** | **≤0.015 (S)** | **≥128/4 (R)** | **≥8/152 (R)** | **≥ 3 2 (R)** | **≥64 (R)** | **≥ 6 4 (R)** | **≥64 (R)** | **≥ 8 (R)** | **≥32/16 (R)** | **≤0.06 (S)** | **≤ 4 (S)** | **≥ 3 2 (R)** |
| **k7 6** | **≥128 (R)** | **≥ 3 2 (R)** | **≥16 (R)** | **≥3 2 (R)** | **1 (S)** | **≥ 1 6 (R)** | **≥128/4 (R)** | **≥8/152 (R)** | **≥ 3 2 (R)** | **≥64 (R)** | **≥ 6 4 (R)** | **≥64 (R)** | **≥ 8 (R)** | **≥32/16 (R)** | **≥ 3 2 (R)** | **≥64 (R)** | **≥ 3 2 (R)** |
| **k7 7** | **≥128 (R)** | **≥ 3 2 (R)** | **≤ 1 (S)** | **≥3 2 (R)** | **≤ 0.2 5 (S)** | **≤0.015 (S)** | **>128/4 (R)** | **≤0.5/9.5 (S)** | **≥ 3 2 (R)** | **≥64 (R)** | **≥ 6 4 (R)** | **≥64 (R)** | **≥ 8 (R)** | **≥32/16 (R)** | **≤0.06 (S)** | **≤ 4 (S)** | **≥ 3 2 (R)** |
| **k7 8** | **≤16 (S)** | **≥ 3 2 (R)** | **≤ 1 (S)** | **≥3 2 (R)** | **≤ 0.2 5 (S)** | **≤0.015 (S)** | **≤ 4 / 4 (S)** | **≤0.5/9.5 (S)** | **≥ 3 2 (R)** | **≥64 (R)** | **≥ 6 4 (R)** | **≤ 8 (S)** | **≤0.06 (S)** | **≤ 8 / 4 (S)** | **≤0.06 (S)** | **≤ 4 (S)** | **≥ 3 2 (R)** |
| **k7 9** | **≥128 (R)** | **≥ 3 2 (R)** | **≥16 (R)** | **≥3 2 (R)** | **1 6 (R)** | **≥ 1 6 (R)** | **≥128/4 (R)** | **≥8/152 (R)** | **≥ 3 2 (R)** | **≥64 (R)** | **≥ 6 4 (R)** | **≥64 (R)** | **≥ 8 (R)** | **≥32/16 (R)** | **≥ 3 2 (R)** | **≥64 (R)** | **≥ 3 2 (R)** |
| **k8 0** | **≥128 (R)** | **≥ 3 2 (R)** | **≥16 (R)** | **≥3 2 (R)** | **1 6 (R)** | **≥ 1 6 (R)** | **≥128/4 (R)** | **≥8/152 (R)** | **≥ 3 2 (R)** | **≥64 (R)** | **≥ 6 4 (R)** | **≥64 (R)** | **≥ 8 (R)** | **≥32/16 (R)** | **≥ 3 2 (R)** | **≥64 (R)** | **≥ 3 2 (R)** |
| **k8 1** | **≥128 (R)** | **≤ 2 (S)** | **≤ 1 (S)** | **≤ 8 (S)** | **≤ 0.2 5 (S)** | **≤0.015 (S)** | **≤ 4 / 4 (S)** | **≤0.5/9.5 (S)** | **2 (S)** | **8 (S)** | **1 (S)** | **≤ 8 (S)** | **≤0.06 (S)** | **≤ 8 / 4 (S)** | **≤0.06 (S)** | **≤ 4 (S)** | **4 (S)** |
| **k8 2** | **≥128 (R)** | **≥ 3 2 (R)** | **≤ 1 (S)** | **≥3 2 (R)** | **≤ 0.2 5 (S)** | **≤0.015 (S)** | **≥128/4 (R)** | **≥8/152 (R)** | **> 3 2 (R)** | **≥64 (R)** | **≥ 6 4 (R)** | **≥64 (R)** | **≥ 8 (R)** | **≥32/16 (R)** | **≤0.06 (S)** | **≤ 4 (S)** | **≥ 3 2 (R)** |
| **k8 3** | **3 2 (S)** | **≤ 2 (S)** | **≤ 1 (S)** | **≥3 2 (R)** | **≤ 0.2 5 (S)** | **≤0.015 (S)** | **≤ 4 / 4 (S)** | **≤0.5/9.5 (S)** | **2 (S)** | **8 (S)** | **1 (S)** | **≤ 8 (S)** | **≥ 8 (R)** | **≤ 8 / 4 (S)** | **≤0.06 (S)** | **≤ 4 (S)** | **4 (S)** |
| **k8 4** | **3 2 (S)** | **≤ 2 (S)** | **≤ 1 (S)** | **≤ 8 (S)** | **≤ 0.2 5 (S)** | **≤0.015 (S)** | **≤ 4 / 4 (S)** | **≤0.5/9.5 (S)** | **≤ 0.1 2 (S)** | **≤ 4 (S)** | **≤ 0.1 2 (S)** | **≤ 8 (S)** | **≤0.06 (S)** | **≤ 8 / 4 (S)** | **≤0.06 (S)** | **≤ 4 (S)** | **≤ 0.5 (S)** |
| **k8 5** | **≥128 (R)** | **≥ 3 2 (R)** | **≤ 1 (S)** | **≥3 2 (R)** | **≤ 0.2 5 (S)** | **≤0.015 (S)** | **≥128/4 (R)** | **≥8/152 (R)** | **≥ 3 2 (R)** | **≥64 (R)** | **≥ 6 4 (R)** | **≥64 (R)** | **0. 1 2 (S)** | **≥32/16 (R)** | **≤0.06 (S)** | **≤ 4 (S)** | **≥ 3 2 (R)** |
| **k8 6** | **≥128 (R)** | **≥ 3 2 (R)** | **≥16 (R)** | **≥3 2 (R)** | **≥ 3 2 (R)** | **≥ 1 6 (R)** | **≥128/4 (R)** | **≥8/152 (R)** | **≥ 3 2 (R)** | **≥64 (R)** | **≥ 6 4 (R)** | **≥64 (R)** | **≥ 8 (R)** | **≥32/16 (R)** | **≥ 3 2 (R)** | **≥64 (R)** | **≥ 3 2 (R)** |
| **k8 7** | **≥128 (R)** | **≥ 3 2 (R)** | **≥16 (R)** | **≥3 2 (R)** | **≤ 0.2 5 (S)** | **≤0.015 (S)** | **≤ 4 / 4 (S)** | **≥8/152 (R)** | **≥ 3 2 (R)** | **≥64 (R)** | **≥ 6 4 (R)** | **≥64 (R)** | **≥ 8 (R)** | **≤ 8 / 4 (S)** | **≤0.06 (S)** | **≥64 (R)** | **≥ 3 2 (R)** |
| **k8 8** | **≥128 (R)** | **≥ 3 2 (R)** | **≤ 1 (S)** | **≥3 2 (R)** | **≤ 0.2 5 (S)** | **0.5 (S)** | **≥128/4 (R)** | **≥8/152 (R)** | **≥ 3 2 (R)** | **≥64 (R)** | **≥ 6 4 (R)** | **≥64 (R)** | **1 (S)** | **≥32/16 (R)** | **≤0.06 (S)** | **≤ 4 (S)** | **≥ 3 2 (R)** |
| **k8 9** | **≥128 (R)** | **≥ 3 2 (R)** | **≥16 (R)** | **≥3 2 (R)** | **≥ 3 2 (R)** | **≥ 1 6 (R)** | **≥128/4 (R)** | **≥8/152 (R)** | **≥ 3 2 (R)** | **≥64 (R)** | **≥ 6 4 (R)** | **≥64 (R)** | **≥ 8 (R)** | **≥32/16 (R)** | **≥ 3 2 (R)** | **≥64 (R)** | **≥ 3 2 (R)** |
| **k9 0** | **≥128 (R)** | **≥ 3 2 (R)** | **≥16 (R)** | **≥3 2 (R)** | **≥ 3 2 (R)** | **≥ 1 6 (R)** | **≥128/4 (R)** | **≥8/152 (R)** | **≥ 3 2 (R)** | **≥64 (R)** | **≥ 6 4 (R)** | **≥64 (R)** | **≥ 8 (R)** | **≥32/16 (R)** | **≥ 3 2 (R)** | **≥64 (R)** | **≥ 3 2 (R)** |
| **k9 1** | **≥128 (R)** | **≥ 3 2 (R)** | **≥16 (R)** | **≥3 2 (R)** | **≤ 0.2 5 (S)** | **≤0.015 (S)** | **≥128/4 (R)** | **≥8/152 (R)** | **≥ 3 2 (R)** | **≥64 (R)** | **≥ 6 4 (R)** | **≥64 (R)** | **≥ 8 (R)** | **≥32/16 (R)** | **≤0.06 (S)** | **≤ 4 (S)** | **≥ 3 2 (R)** |
| **k9 2** | **≥128 (R)** | **≥ 3 2 (R)** | **≥16 (R)** | **≥3 2 (R)** | **≤ 0.2 5 (S)** | **0.5 (S)** | **≥128/4 (R)** | **≤0.5/9.5 (S)** | **≥ 3 2 (R)** | **≥64 (R)** | **≥ 6 4 (R)** | **≤ 8 (S)** | **≥ 8 (R)** | **≥32/16 (S)** | **≤0.06 (S)** | **≥64 (R)** | **≥ 3 2 (R)** |
| **k9 3** | **≤16 (S)** | **≥ 3 2 (R)** | **≤ 1 (S)** | **≥3 2** **(R)** | **≤ 0.2 5 (S)** | **≤0.015 (S)** | **≤ 4 / 4 (S)** | **≤0.5/9.5 (S)** | **≥ 3 2 (R)** | **≥64 (R)** | **≥ 6 4 (R)** | **≥64 (R)** | **≥ 8 (R)** | **≤ 8 / 4 (S)** | **≤0.06 (S)** | **≤ 4 (S)** | **≥ 3 2 (R)** |
| **k9 4** | **≥128 (R)** | **≥ 3 2 (R)** | **≥16 (R)** | **≥3 2 (R)** | **≤ 0.2 5 (S)** | **≤0.015 (S)** | **≥128/4 (R)** | **≤0.5/9.5 (S)** | **≥ 3 2 (R)** | **≥64 (R)** | **≥ 6 4 (R)** | **≥64 (R)** | **≥ 8 (R)** | **≥32/16 (R)** | **≤0.06 (S)** | **≤ 4 (S)** | **≥ 3 2 (R)** |
| **k9 5** | **3 2 (S)** | **≤ 2 (S)** | **≤ 1 (S)** | **≥3 2 (R)** | **≤ 0.2 5 (S)** | **0.5 (S)** | **≤ 4 / 4 (S)** | **≤0.5/9.5 (S)** | **2 (S)** | **≥64 (R)** | **1 (S)** | **≤ 8 (S)** | **≤0.06 (S)** | **≤ 8 / 4 (S)** | **≤0.06 (S)** | **≤ 4 (S)** | **4 (S)** |
| **k9 6** | **≥128 (R)** | **≥ 3 2 (R)** | **≤ 1 (S)** | **≥3 2 (R)** | **≤ 0.2 5 (S)** | **0.5 (S)** | **≥128/4 (R)** | **≥8/152 (R)** | **≥ 3 2 (R)** | **≥64 (R)** | **≥ 6 4 (R)** | **≥64 (R)** | **≥ 8 (R)** | **≥32/16 (R)** | **≤0.06 (S)** | **≤ 4 (S)** | **≥ 3 2 (R)** |
| **k9 7** | **3 2 (S)** | **≤ 2 (S)** | **≤ 1 (S)** | **≥3 2 (R)** | **≤ 0.2 5 (S)** | **≤0.015 (S)** | **≤ 4 / 4 (S)** | **≥8/152 (R)** | **2 (S)** | **8 (S)** | **1 (S)** | **≤ 8 (S)** | **≤0.06 (S)** | **≥32/16 (R)** | **≤0.06 (S)** | **≤ 4 (S)** | **4 (S)** |
| **k9 8** | **≤16 (S)** | **≥ 3 2 (R)** | **≤ 1 (S)** | **≥3 2 (R)** | **≤ 0.2 5 (S)** | **0.5 (S)** | **≥128/4 (R)** | **≥8/152 (R)** | **≥ 3 2 (R)** | **≥64 (R)** | **≥ 6 4 (R)** | **≥64 (R)** | **2 (S)** | **≥32/16 (R)** | **≤0.06 (S)** | **≤ 4 (S)** | **≥ 3 2 (R)** |
| **k9 9** | **≥128 (R)** | **≥ 3 2 (R)** | **≤ 1 (S)** | **≥3 2 (R)** | **≤ 0.2 5 (S)** | **0.5 (S)** | **≥128/4 (R)** | **≤0.5/9.5 (S)** | **≥ 3 2 (R)** | **≥64 (R)** | **≥ 6 4 (R)** | **≥64 (R)** | **2 (S)** | **≥32/16 (R)** | **≤0.06 (S)** | **≤ 4 (S)** | **≥ 3 2 (R)** |
| **k100** | **≤16 (S)** | **≥ 3 2 (R)** | **≤ 1 (S)** | **≥3 2 (R)** | **≤ 0.2 5 (S)** | **0.5 (S)** | **≥128/4 (R)** | **≥8/152 (R)** | **≥ 3 2 (R)** | **≥64 (R)** | **≥ 6 4 (R)** | **≥64 (R)** | **2 (S)** | **≥32/16 (R)** | **≤0.06 (S)** | **≤ 4 (S)** | **≥ 3 2 (R)** |

The results are expressed as resistant (R) or sensitive (S) according to the Clinical and Laboratory Standards Institute (CLSI) interpretive criteria against 100 isolates of *Klebsiella pneumoniae* (*K. pneumoniae*).

**F:** Nitrofurantoin**, CZ:** Cefazolin**, CN:** Gentamycin**, AMP:** Ampicillin**, IPM:** Imipenem**, ERT:** Ertapenem**, TZP:** Tazobactam/piperacillin**, SXT:** Sulfamethoxazole /Trimethoprim**, FEP:** Cefepime**, CXM:** Cefuroxime**, CTX:** Cefotaxime, **FOX:** Cefoxitin, **LEV:** Levofloxacin, **SAM:** Ampicillin/Sulbactam, **MEM:** Meropenem, **AK:** Amikacin, **CAZ:** Ceftazidime.
